# Integration of Spatial Transcriptomics and Mendelian Randomization Identifies Candidate Molecular Regulators of Endometrial Cancer Progression

**DOI:** 10.3390/cells15171559

**Published:** 2026-08-28

**Authors:** Jianan Zhao, Xiaonan Liu, Huiyang Zhao, Pingping Zhang, Shenxin Wang, Congying Duan, Yue Liu, Wei Wang, Ping Jiao, Jie Ma

**Affiliations:** 1 School of Pharmaceutical Sciences, Jilin University, Changchun 130021, China; zhaojn21@mails.jlu.edu.cn (J.Z.); lxn24@mails.jlu.edu.cn (X.L.); huiyang24@mails.jlu.edu.cn (H.Z.); zhangpp24@mails.jlu.edu.cn (P.Z.); wsx25@mails.jlu.edu.cn (S.W.); duancy25@mails.jlu.edu.cn (C.D.); yue_liu25@mails.jlu.edu.cn (Y.L.); wangwei970822@jlu.edu.cn (W.W.); 2 Key Laboratory of Pathobiology, Ministry of Education, Jilin University, Changchun 130021, China

**Keywords:** endometrial cancer, spatial transcriptomics, cell proliferation

## Abstract

**Highlights:**

**What are the main findings?**

- MM0 was identified as a putative stemness-associated malignant epithelial transcriptional subpopulation, and MM0-enriched regions showed higher inferred JAK–STAT and hypoxia scores in the cross-sectional spatial analysis.
- Integrative Mendelian randomization and colocalization analyses prioritized three candidate genes: *DNAJA4*, *HSPA6*, and *LMNA*. Subsequent experiments showed that knockdown of either *DNAJA4* or *HSPA6* suppressed estrogen-stimulated cell proliferation and migration, with reduced CDK1 and Cyclin B expression.

**What are the implications of the main findings?**

- These findings illustrate a multi-omics approach linking malignant epithelial heterogeneity and spatial tumor biology to genetically supported candidate genes associated with endometrial cancer risk.
- *DNAJA4* and *HSPA6* warrant further validation as candidate functional genes in endometrial cancer and may inform future studies of therapeutic strategies.

**Abstract:**

Endometrial cancer (EC) is a common gynecologic malignancy arising from the epithelial cells of the endometrium. The marked cellular heterogeneity of EC and features of its tumor immune microenvironment (TIME) contribute to disease complexity and have been associated with poor prognosis. This study integrates single-cell RNA sequencing, spatial transcriptomics, and Mendelian randomization (MR) to identify candidate genes associated with EC. Single-cell analysis identified MM0 as a putative stemness-associated transcriptional subpopulation with the highest CytoTRACE-inferred score; irGSEA indicated enrichment of proliferation- and stress-response pathways. Spatial data were analyzed with RCTD, MISTy, and stLearn to estimate spatial associations and pathway activities. MR and colocalization analyses integrating eQTL data and EC GWAS summary statistics prioritized DNAJA4, HSPA6, and LMNA as candidate genes with potential causal associations with EC risk. The expression patterns of these candidate genes were further examined in patient-derived samples. In Ishikawa cells cultured under high-estrogen conditions, siRNA-mediated knockdown of DNAJA4 and HSPA6 significantly suppressed proliferation and migration, accompanied by reduced CDK1 and Cyclin B expression. Collectively, these findings provide insight into EC heterogeneity and support further mechanistic investigation of candidate genes associated with malignant epithelial proliferation.

## 1. Introduction

Endometrial cancer (EC) is one of the most common gynecologic malignancies, and its incidence has increased alongside socioeconomic development. This trend contributes to a growing disease burden, and women face a substantial lifetime risk of developing EC [1,2]. Current cancer treatment strategies encompass surgery, radiotherapy, chemotherapy, endocrine therapy, targeted therapy, and immunotherapy, with increasing emphasis on individualized treatment according to tumor biology and molecular characteristics [3]. Although early-stage EC can generally be managed effectively with surgery, advanced and recurrent disease remain difficult to treat and contribute to the ongoing disease burden [1,4,5]. One factor contributing to these treatment challenges is the marked molecular and cellular heterogeneity of EC [6]. This heterogeneity is associated with uncontrolled epithelial proliferation and a dynamic hormonal microenvironment, both of which may contribute to the variable clinical course of EC [7,8]. A clearer understanding of the mechanisms underlying malignant proliferation in EC may therefore inform the development of more individualized treatment strategies.

EC progression involves the continued expansion of malignant epithelial cells and their complex interactions within the tumor immune microenvironment (TIME) [9,10]. Previous studies have linked tumor-cell survival and rapid proliferation under stressful conditions to several protein families. For example, heat shock proteins and their co-chaperones have been implicated in immune recognition, whereas lamins can influence cellular mechanical plasticity; both may be relevant to tumor progression. Recent advances in single-cell and spatial transcriptomic technologies have expanded the ability to investigate complex tumor biology and characterize the tumor ecosystem [11,12,13]. Single-cell RNA sequencing (scRNA-Seq) enables the mapping of cellular subpopulations and inference of cell-state differentiation trajectories [11,12]. Spatial transcriptomics provides architectural context for estimating within-section cellular distributions and associations [13]. By using genetic variants as instrumental variables, MR can reduce bias from confounding and reverse causation in observational studies, thereby enabling the evaluation of potential causal associations between genetically predicted gene expression and disease risk [14].

Although extensive bulk genomic and transcriptomic profiling has established broad molecular classifications for EC, the spatial and single-cell characteristics of malignant epithelial proliferation remain underexplored. Traditional bulk sequencing may obscure putative stemness-associated transcriptional subpopulations linked to tumor-related phenotypes. Integrating complementary omics layers may identify cross-layer regulatory relationships that are missed by single-omics analyses and provide a broader basis for prioritizing cancer-associated candidate genes and characterizing tumor-related phenotypes [15]. Furthermore, a persistent challenge in multi-omics research is distinguishing genes whose expression changes merely accompany disease from those with potential causal or functional relevance. Single-modality analytical approaches are limited in their ability to evaluate potential causal associations because they do not integrate high-resolution spatial and single-cell data with genetic evidence and functional assays. Consequently, the relationships among candidate-gene expression, stemness-related transcriptional programs in malignant epithelial cells, and immune-infiltration patterns in EC remain incompletely characterized. This gap limits the biological interpretation and potential clinical translation of transcriptomic findings.

To address these gaps, we hypothesize that distinct candidate-gene expression patterns are associated with stemness-related transcriptional features, cell-cycle activity, spatial features, and immune-infiltration patterns in EC. This study uses an integrative, multilayered framework combining scRNA-Seq, spatial transcriptomics, and MR to prioritize genetically supported candidate genes for functional assessment. We then evaluate spatially inferred pathway activities and the associations of candidate-gene expression with inferred immune-infiltration patterns in the TIME, clinical outcomes, and predicted drug sensitivity. Finally, we examine the effects of the selected candidate genes on epithelial proliferation and the expression of cell-cycle-related proteins under high-estrogen conditions.

## 2. Materials and Methods

### 2.1. Patient Materials

Fresh tissue samples were collected at the Second Hospital of Jilin University between May and October 2025. Independent EC specimens were obtained from patients undergoing surgical resection, and four independent normal endometrial control specimens were obtained from individuals undergoing hysteroscopic surgery. The tumor and control specimens were not paired. Written informed consent was obtained from all participants before tissue collection.

### 2.2. Reagents and Materials

The Ishikawa cell line (RRID: CVCL_2529) was obtained from HyCyte™ Biosciences (Suzhou, China). The cell line was validated via STR profiling within the last three years and confirmed to be free of mycoplasma contamination. Antibodies were procured from HuaBio (Hangzhou, China) and Servicebio (Wuhan, China), and 17β-estradiol (E2) was obtained from KKL Med Inc. (Ashland, VA, USA). Small interfering RNAs (siRNAs) targeting *DNAJA4*, *HSPA6*, and *LMNA* were synthesized by Sangon Biotech (Shanghai, China). A validated non-targeting siRNA served as the negative control (NC). The sense sequences were as follows: si-*HSPA6*, 5′-GCUGUGACUGUCAGGGCUAUG-3′; si-*DNAJA4*, 5′-CGACAGAAAGUGAGGAUUACA-3′; and si-LMNA, 5′-GAACUGCAGCAUCAUGUAAUC-3′.

### 2.3. Data Acquisition

1. GEO Database: Single-cell RNA sequencing (scRNA-Seq) data from five tumor samples (GSE173682) and one spatial transcriptomic tissue section (GSM6177623) were downloaded from the Gene Expression Omnibus (GEO) database (https://www.ncbi.nlm.nih.gov/geo/info/datasets.html; accessed on 20 April 2025) for subsequent analyses. Available clinicopathological information for the spatial transcriptomic sample and the single-cell reference samples is provided in Table S1.
2. TCGA Database: Raw gene expression profile data from 589 endometrial cancer-related samples were obtained from The Cancer Genome Atlas (TCGA) database, including 554 tumor samples and 35 normal control samples.
3. Exposure Data: Expression quantitative trait locus (eQTL) data were obtained from the eQTLGen Consortium and were generated on the basis of a large-scale meta-analysis of genome-wide blood gene expression data.
4. Outcome Data: Outcome-related summary statistics from the genome-wide association study (GWAS) were obtained from the European Bioinformatics Institute database (accession number: ebi-a-GCST90018838). The study population was primarily of European ancestry and included 2188 patients with EC and 237,839 healthy controls.

### 2.4. Single-Cell Data Quality Control

The raw expression matrix was preprocessed using the Seurat R package (v5.3.0). Cell filtering was performed based on total unique molecular identifier (UMI) counts, the number of detected genes, and the proportions of mitochondrial and ribosomal transcripts in each cell. Outliers were defined as observations exceeding three times the median absolute deviation (MAD). To exclude potential doublets, cells with abnormally high UMI counts or numbers of detected genes were removed. Cells with elevated proportions of mitochondrial transcripts were also excluded, as these are generally indicative of low-quality or apoptotic cells. Subsequently, DoubletFinder (v2.0.4) was applied to each sample to systematically identify and remove residual doublets.

### 2.5. Data Normalization and Dimensionality Reduction

Data normalization was performed using the NormalizeData function, and highly variable genes were identified with FindVariableFeatures. Subsequently, the data were scaled using ScaleData, with mitochondrial and ribosomal gene expression levels as well as cell cycle scores, calculated by CellCycleScoring, regressed out to minimize potential confounding effects. Principal component analysis (PCA) was then conducted for linear dimensionality reduction using RunPCA. To further correct for batch effects, the Harmony algorithm was applied in the PCA embedding space. For visualization, non-linear dimensionality reduction was carried out using Uniform Manifold Approximation and Projection (UMAP) through RunUMAP. Cell clustering was performed with FindNeighbors and FindClusters. Cell types were annotated on the basis of canonical marker genes curated from the literature and the CellMarker database, with further refinement using automated annotation in the SingleR package (v2.10.0).

### 2.6. irGSEA Enrichment Analysis

Individual cells were independently scored using AUCell, UCell, singscore, and ssGSEA. Differentially enriched gene sets for each cluster were evaluated across these enrichment matrices, with significance defined as an adjusted *p* < 0.05 (Wilcoxon rank-sum test). A robust rank aggregation algorithm was employed to identify statistically significant gene sets that demonstrated consistent enrichment directionality across all four scoring methods.

### 2.7. Pseudotime Trajectory Analysis

The Monocle package (v2.36.0) was used to infer cell-state trajectories by ordering asynchronous individual cells along a transcriptionally defined pseudotime continuum.

### 2.8. RCTD Deconvolution

Robust Cell-Type Decomposition (RCTD) was employed to map cell types onto spatial transcriptomic spots [16]. Utilizing annotated scRNA-Seq profiles as a reference, RCTD decomposes mixed RNA signals at the pixel level while statistically accounting for cross-platform batch effects (platform effects) between single-cell and spatial sequencing technologies.

### 2.9. Spatial Cellular Associations

The MISTy (Multiview Intercellular SpaTial modeling) pipeline was used to estimate intracellular and intercellular associations across different spatial contexts [17]. It estimates the contribution of multiple spatial views to the expression of selected markers. View-specific importance scores were interpreted as model-derived associations rather than direct physical interactions.

### 2.10. Spatial Trajectory Inference via stLearn

Spatial trajectory and clustering analyses were performed using the stLearn Python package (v0.4.12). The raw spatial transcriptomics data were preprocessed and normalized to reduce technical variation. Subsequently, principal component analysis and neighborhood graph construction were performed, and the Leiden clustering method was applied to identify spatially resolved cell populations and map their distribution within the tissue. To improve the robustness of spatial clustering, clustering results were evaluated across different resolution parameters and compared with the spatial distribution of histological regions and representative marker genes. The final clustering resolution was selected based on cluster stability, spatial continuity, and biological interpretability. These analyses were used to support spatial localization and exploratory biological interpretation rather than to define an independently validated spatial subtype.

### 2.11. Mendelian Randomization (MR) Analysis

Single-nucleotide polymorphisms (SNPs) significantly associated with expression at the target gene locus were selected as potential instrumental variables (IVs). For cis-eQTL instruments, SNPs located within the predefined cis-regulatory window of each gene were prioritized. To ensure independence among the IVs, linkage disequilibrium (LD) clumping was performed using R^2^ < 0.001 and a window size of 10,000 kb. The strength of each selected instrument was evaluated using the F statistic, calculated as F = beta^2^/SE^2^, and SNPs with F statistics ≤ 10 were excluded to minimize weak-instrument bias. The inverse-variance weighted (IVW) method was used as the primary approach to estimate potential causal associations when multiple independent IVs were available; when only a single SNP was available, the Wald ratio method was applied. Complementary methods, including MR-Egger, the weighted median method, and the weighted mode method, were also used when applicable to assess the consistency of effect direction and reduce the influence of potential invalid instruments. The MR results were interpreted as genetic evidence supporting potential causal associations between gene expression and EC risk, rather than as definitive proof of direct biological causality.

### 2.12. Sensitivity Analysis and Heterogeneity Testing

Leave-one-out sensitivity analysis was performed to assess whether the overall MR estimate was disproportionately influenced by any highly influential single-nucleotide polymorphism (SNP). Heterogeneity among the selected SNPs was evaluated using Cochran’s Q statistic, with *p* < 0.05 considered indicative of significant heterogeneity. When multiple IVs were available, potential horizontal pleiotropy was further assessed using the MR-Egger intercept test. Concordance across IVW, MR-Egger, weighted median, weighted mode, heterogeneity testing, and leave-one-out analysis was considered to provide additional support for the MR signal. For genes supported by only a limited number of instruments, the results were interpreted cautiously and were further integrated with colocalization, transcriptomic, spatial, and experimental evidence.

### 2.13. Colocalization Analysis

Colocalization analysis was performed using the coloc R package (v5.2.3) to assess whether expression quantitative trait locus (eQTL) signals and EC genome-wide association study (GWAS) signals are consistent with a shared causal variant. The analysis was restricted to a 100-kb genomic window upstream and downstream of the index single-nucleotide polymorphism (SNP). Strong evidence of colocalization was defined as PP.H4 > 0.90, indicating support for a shared-variant model.

### 2.14. Bioinformatic Profiling of Tumor Immune Infiltration

The CIBERSORT algorithm, based on support vector regression utilizing a 547-gene signature matrix (LM22), was applied to deconvolve bulk expression profiles into the relative proportions of 22 distinct immune-infiltrating cell phenotypes. Subsequent correlation analyses were performed between prioritized candidate-gene expression and the estimated fractions of these immune-cell populations.

### 2.15. Construction of a Nomogram Model

A nomogram was constructed from a multivariable Cox regression model integrating candidate-gene expression signatures and relevant clinical parameters to estimate clinical outcomes. A risk score was first derived from the expression pattern of candidate genes and then combined with available clinical variables, including age and tumor grade, to construct the final nomogram for predicting 1-, 3-, and 5-year overall survival. The predictive performance of the nomogram was evaluated using time-dependent receiver operating characteristic (ROC) curves and the corresponding area under the curve (AUC). Calibration curves were generated to compare the predicted survival probabilities with the observed outcomes. Decision curve analysis (DCA) was further performed to assess the potential clinical net benefit of the nomogram across different threshold probabilities. These internal performance assessments were used to evaluate the discrimination, calibration, and potential clinical utility of the model.

### 2.16. Drug Sensitivity Prediction

As an exploratory analysis, drug sensitivity (estimated as half-maximal inhibitory concentration, IC50) for chemotherapeutic agents was predicted using the calcPhenotype function within the oncoPredict R package (v1.2), leveraging reference data from the Genomics of Drug Sensitivity in Cancer (GDSC) database. Raw expression data were preprocessed using the default parameters, with the ‘combat’ method applied for batch correction and duplicate gene-expression values averaged before prediction.

### 2.17. Statistical Analysis

All bioinformatic and statistical analyses were performed using R software (version 4.3.0) and Python (version 3.8). For MR analysis, the three core assumptions of relevance, independence, and exclusion restriction were considered during instrument selection and sensitivity assessment. Instrument strength was assessed using the F statistic, and weak instruments were excluded. For nomogram construction, model performance was assessed using ROC curves, calibration analysis, and DCA. For spatial clustering, robustness was evaluated by considering clustering stability under different parameters together with spatial continuity and marker-gene consistency. All statistical tests were two-sided, and a *p*-value < 0.05 was considered statistically significant unless otherwise specified.

## 3. Results

### 3.1. Quality Control and Single-Cell Data Pre-Processing

During quality control, cells identified as outliers or expressing fewer than 200 genes were removed. After doublets were removed, 28,754 high-quality cells remained. Violin plots and scatter plots were generated from the filtered data (Figure S1A,B). Subsequently, 2000 highly variable genes were identified (Figure S1C). The data were normalized, scaled, subjected to PCA, and corrected for batch effects using Harmony (Figure S1D–F).

### 3.2. Data Standardization, Cell Annotation, and Identification of Differentially Expressed Genes in Malignant Cells

**Figure 1:**
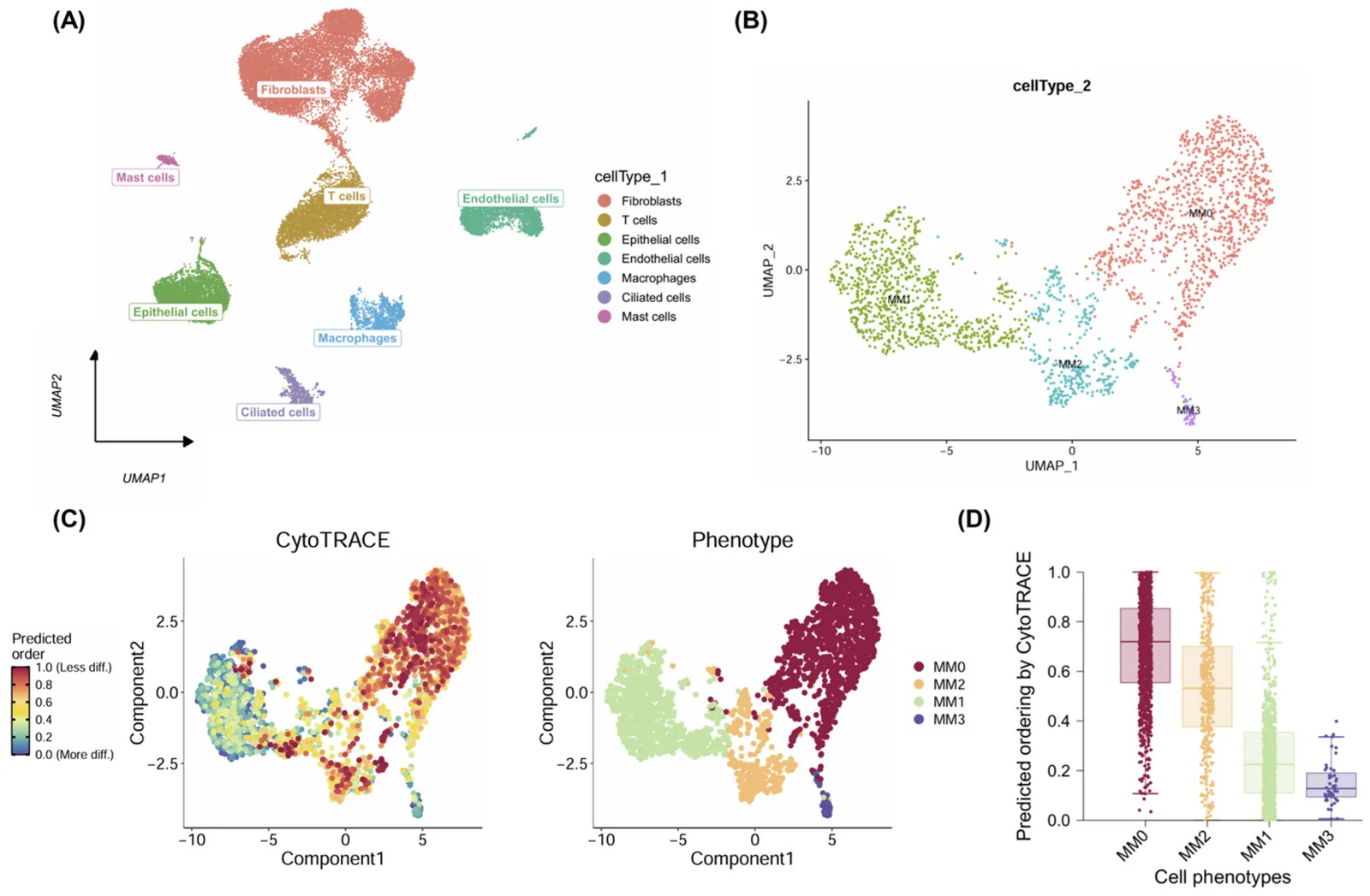
Single-cell landscape and identification of malignant epithelial subpopulations in EC. (**A**) UMAP visualization of 28,754 high-quality cells from EC samples, color-coded by seven major cell types. (**B**) UMAP embedding of malignant epithelial cells, subdivided into four clusters (MM0–MM3). (**C**) CytoTRACE-based estimates of relative differentiation potential and stemness-associated transcriptional features. Red indicates a higher inferred stemness-associated score, whereas blue indicates greater inferred differentiation. (**D**) Comparison of CytoTRACE-inferred scores across MM0–MM3, designating MM0 as a putative stemness-associated transcriptional subpopulation.

Following dimensionality reduction by UMAP, 11 distinct subpopulations were identified (Figure S1G). Using established marker genes, the 11 subpopulations were assigned to seven major cell categories: fibroblasts, T cells, epithelial cells, endothelial cells, macrophages, ciliated cells, and mast cells (Figure 1A). Expression patterns of key marker genes across the seven cell types were visualized using a dot plot (Figure S2A). Cell-type proportions across different samples were displayed in a bar plot (Figure S2B). The epithelial cell population was further subset for secondary clustering (Figure S2C). Genomic copy number variations were subsequently inferred from the scRNA-Seq data using the CopyKAT (Copy number Karyotyping of Tumors) package to distinguish malignant from non-malignant cells by identifying aneuploid cells. Of the 3884 epithelial cells analyzed, 2266 were classified as malignant and 1618 as non-malignant (Figure S2D).

### 3.3. Secondary Clustering and CytoTRACE Assessment of Stemness-Associated Transcriptional States in Malignant Epithelial Cells

Malignant cells were further extracted for secondary clustering, yielding four subpopulations designated MM0, MM1, MM2, and MM3 (Figure 1B). Cell-type proportions of the four subpopulations across different samples were visualized in bar plots (Figure S2E). Differential gene expression among the four subpopulations was identified using the FindAllMarkers function. Using the criteria of adjusted *p*-value < 0.05 and absolute average log2 fold change > 0.585, 293 MM0-specific differentially expressed genes were identified. Next, CytoTRACE (Cellular Trajectory Reconstruction Analysis using gene Counts and Expression) was applied to estimate relative differentiation potential and stemness-associated transcriptional features (Figure 1C,D). MM0 had the highest CytoTRACE score and was therefore designated a putative stemness-associated transcriptional subpopulation. Within the inferred cell-state differentiation trajectory, MM0 was positioned as a transcriptionally inferred early cell state. This computational ordering reflects transcriptional similarities among cells sampled cross-sectionally and does not establish temporal succession, lineage ancestry, or clonal evolution in vivo. Proliferation-related module scoring further indicated that MM0 exhibited the strongest proliferation-associated transcriptional activity among the malignant epithelial subpopulations (Figure S3A–C).

### 3.4. Cluster-Specific Hallmark Pathway Enrichment Revealed by irGSEA

**Figure 2:**
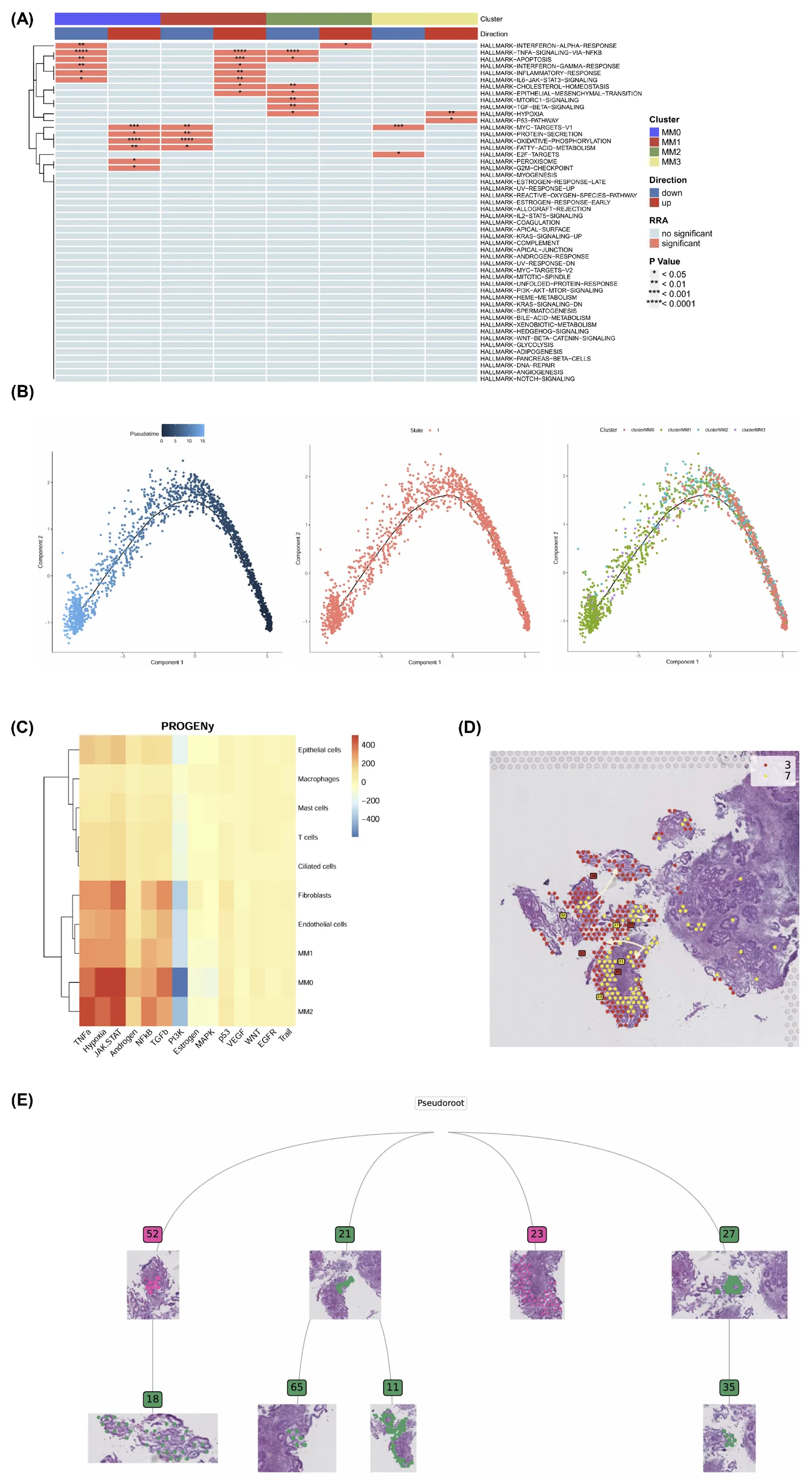
Pathway activity, pseudotemporal dynamics, and spatial organization of malignant epithelial subpopulations in EC. (**A**) Heatmap of irGSEA scores for Hallmark pathways across malignant epithelial subpopulations, with statistical significance indicated by asterisks. (**B**) Monocle-based pseudotime ordering showing an inferred cell-state differentiation trajectory spanning MM0–MM3. (**C**) PROGENy-inferred pathway activity scores in the spatial transcriptomic data, showing higher JAK–STAT and hypoxia scores in MM0-enriched regions. (**D**,**E**) stLearn-based spatial clustering and inferred relationships among transcriptional states.

In this study, the AUCell, UCell, singscore, and ssGSEA algorithms were applied to quantify Hallmark gene-set enrichment scores for individual cells. Four enrichment-score matrices were subsequently used to identify differentially enriched gene sets across cell clusters. Key gene sets identified by Robust Rank Aggregation (RRA) were visualized across cell clusters in a heatmap, with asterisks in the upper triangle of each cell indicating the level of statistical significance (*p*-value). A dendrogram on the left indicates the similarity of gene-set enrichment patterns among clusters. The “Direction” legend (up or down arrows) indicates whether the enrichment score of each gene set is higher or lower in a given cluster relative to the others. For example, the HALLMARK_TNFA_SIGNALING_VIA_NFKB pathway was significantly enriched in the MM0 subpopulation (Figure 2A).

### 3.5. Pseudotime Analysis

Cell similarity was used to infer a cell-state differentiation trajectory. Monocle ordered asynchronous malignant epithelial cells along a transcriptionally defined pseudotime continuum (Figure 2B). Genes showing the most pronounced expression changes along this inferred ordering were selected and grouped into three expression clusters. HLA-C, HLA-B, and HLA-A showed peak expression toward one end of the inferred pseudotime continuum (Figure S4A).

### 3.6. Dimensionality Reduction, Clustering, and RCTD Deconvolution of Spatial Transcriptomic Data

Spatial transcriptomics data were loaded and quality-controlled (Figure S4B), followed by normalization, scaling, PCA, and Bayesian clustering. This process divided the spots into four subgroups (Figure S4C). Deconvolution analysis was performed using the spacexr package by integrating the spatial transcriptomic data with the single-cell reference profiles to estimate cell-type proportions at each spot. Each spot was assigned the cell type with the highest estimated proportion as its dominant cell-type label (Figure S4D). In an exploratory within-section analysis, greater RCTD-derived MM0 weights spatially co-occurred with higher proliferation-related transcriptional scores (Figure S3D–F).

### 3.7. Spatial Association and Pathway Activity Analyses

MISTy was used to estimate intracellular and intercellular associations among marker genes. Following deconvolution, cell-type-specific associations were inferred from the annotated spot identities and visualized as heatmaps and network diagrams (Figure S4E,F). The model indicated an association between the MM0 and MM2 subpopulations. PROGENy was then used to estimate pathway activity scores in the spatial transcriptomic data (Figure 2C). MM0-enriched spots showed higher PROGENy-inferred JAK–STAT and hypoxia pathway scores.

### 3.8. stLearn Spatial Trajectory Inference

The stLearn package was used to examine the spatial distribution of transcriptional states and their inferred relationships. Multiple spatial clusters were identified using the Leiden clustering algorithm. After a root node and a specific cell population were selected, an inferred spatial trajectory tree was generated. This exploratory analysis represented the spatial organization and inferred relationships among transcriptional states in the analyzed tissue section (Figure 2D,E).

### 3.9. Mendelian Randomization Analysis

**Figure 3:**
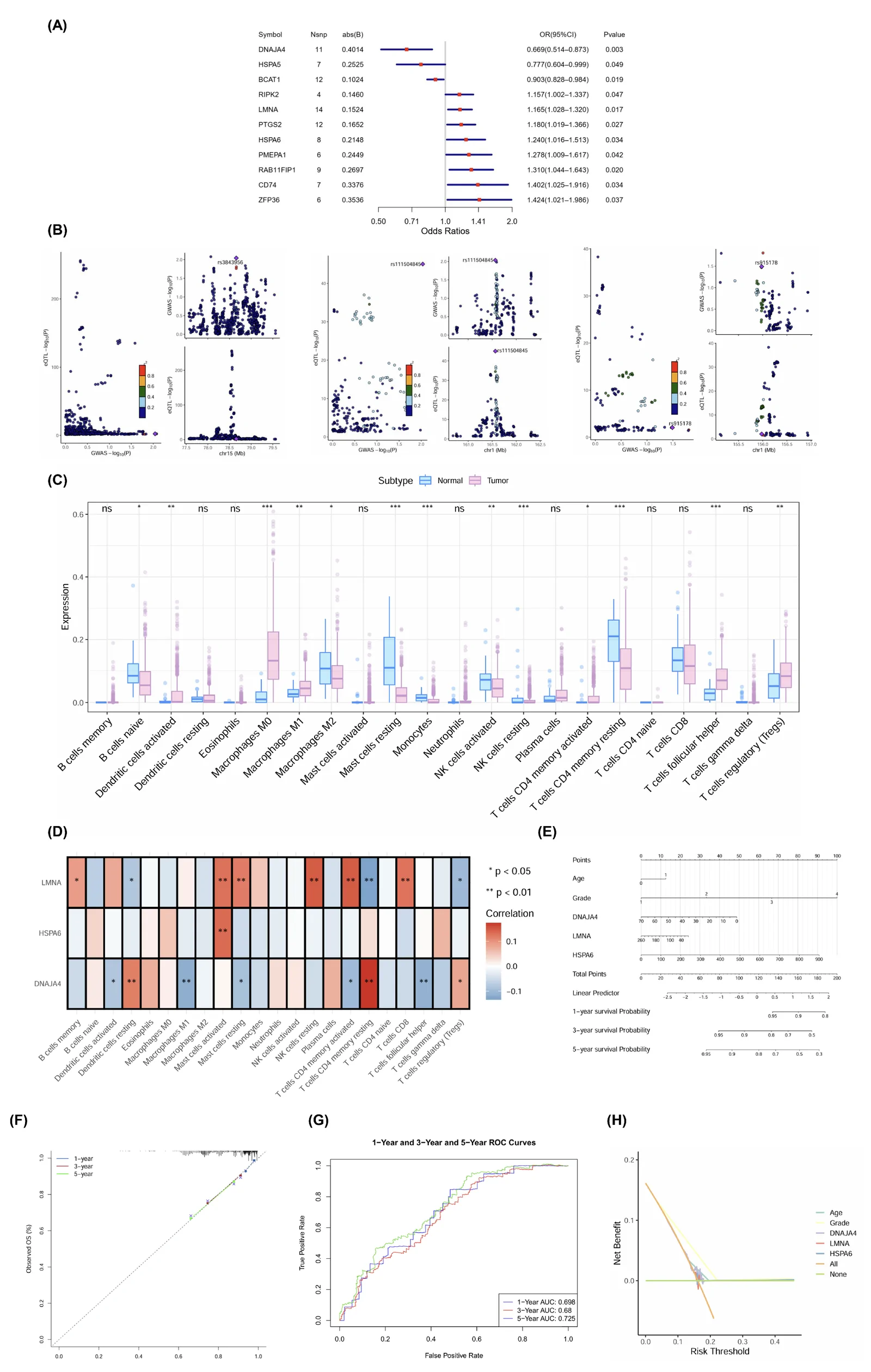
Integrative genetic prioritization, bioinformatic profiling of tumor immune infiltration, and prognostic model construction in EC. (**A**) Forest plot showing the MR estimates for associations between the genetically predicted expression of 11 candidate genes and EC risk, calculated using the inverse-variance weighted (IVW) method. Odds ratios (ORs) > 1 indicate associations with higher EC risk, whereas ORs < 1 indicate associations with lower EC risk. The orange dots represent the estimated odds ratios, and the blue horizontal lines indicate the corresponding 95% confidence intervals. (**B**) LocusCompare plots showing colocalization between whole-blood eQTL and EC GWAS signals for DNAJA4, HSPA6, and LMNA, with PP.H4 > 0.90 indicating strong evidence consistent with a shared causal variant. The purple diamond denotes the lead SNP, whereas the remaining SNPs are colored according to their linkage disequilibrium (LD; r^2^) with the lead SNP. (**C**) Boxplot comparing CIBERSORT-estimated relative immune-cell fractions between normal and tumor tissues. (**D**) Heatmap of Spearman correlations between the expression of DNAJA4, HSPA6, and LMNA and CIBERSORT-estimated fractions of specific immune-cell populations (e.g., Tregs and macrophages). (**E**) A prognostic nomogram integrating gene signatures and clinical parameters (Age, Grade) to predict 1-, 3-, and 5-year overall survival (OS). (**F**–**H**) Internal assessment of nomogram performance using calibration plots (**F**), time-dependent ROC curves (**G**), and decision curve analysis (DCA) (**H**). * *p* < 0.05, ** *p* < 0.01, and *** *p* < 0.001; ns, not significant.

To further prioritize candidate genes with potential causal associations with EC risk among the 293 differentially expressed genes, GWAS summary statistics from 240,027 samples (237,839 controls and 2188 cases; outcome ID: ebi-a-GCST90018838) were utilized. SNPs associated with each gene at a locus-wide significance threshold (*p* < 5 × 10^−5^) were selected as potential instrumental variables, and linkage disequilibrium (LD) was calculated. After LD clumping (R^2^ < 0.001, window size = 10,000 kb), only SNPs with *p* < 5 × 10^−8^ were retained. MR analysis subsequently identified 11 genes whose genetically predicted expression was associated with EC risk (inverse-variance weighted [IVW] *p* < 0.05) (Figure 3A). The 11 genes were *BCAT1*, *CD74*, *DNAJA4*, *HSPA5*, *HSPA6*, *LMNA*, *PMEPA1*, *PTGS2*, *RAB11FIP1*, *RIPK2*, and *ZFP36. BCAT1* (OR = 0.903, 95% CI: 0.828–0.984, *p* = 0.019), *DNAJA4* (OR = 0.669, 95% CI: 0.514–0.873, *p* = 0.003), and *HSPA5* (OR = 0.777, 95% CI: 0.604–0.999, *p* = 0.049) were associated with a reduced risk. In contrast, *CD74* (OR = 1.402, 95% CI: 1.025–1.916, *p* = 0.034), *HSPA6* (OR = 1.240, 95% CI: 1.016–1.513, *p* = 0.034), *LMNA* (OR = 1.165, 95% CI: 1.028–1.320, *p* = 0.017), *PMEPA1* (OR = 1.278, 95% CI: 1.009–1.617, *p* = 0.042), *PTGS2* (OR = 1.180, 95% CI: 1.019–1.366, *p* = 0.027), *RAB11FIP1* (OR = 1.310, 95% CI: 1.044–1.643, *p* = 0.020), *RIPK2* (OR = 1.157, 95% CI: 1.002–1.337, *p* = 0.047), and *ZFP36* (OR = 1.424, 95% CI: 1.021–1.986, *p* = 0.037) were associated with an elevated risk. Sensitivity analyses were conducted to evaluate the robustness of the 11 MR associations. Furthermore, leave-one-out sensitivity analysis indicated that no individual SNP disproportionately influenced the overall MR estimates (Figure S5A–K). Heterogeneity testing showed no significant heterogeneity for any of the 11 genes (Table S2). Consequently, *BCAT1*, *CD74*, *DNAJA4*, *HSPA5*, *HSPA6*, *LMNA*, *PMEPA1*, *PTGS2*, *RAB11FIP1*, *RIPK2*, and *ZFP36* were selected as the primary candidate genes for subsequent analyses.

### 3.10. Colocalization and Reverse MR

Colocalization analysis was performed for the 11 genes using coloc to compare the eQTL and GWAS signals. The posterior probability of colocalization (PP.H4) exceeded 0.9 for *DNAJA4*, *HSPA6*, and *LMNA* (Figure 3B). In the reverse MR analysis, summary statistics for endometrial cancer (outcome ID: ebi-a-GCST90018838) served as the exposure, with *DNAJA4*, *HSPA6*, and *LMNA* as the outcomes. Reverse MR analysis found no evidence supporting a reverse association between genetic liability to EC and genetically predicted expression of DNAJA4, HSPA6, or LMNA. Consequently, *DNAJA4*, *HSPA6*, and *LMNA* were prioritized as candidate genes for subsequent integrative analyses and functional assessment.

### 3.11. Bioinformatic Profiling of the Tumor Immune Microenvironment

The TIME comprises fibroblasts, immune cells, extracellular-matrix components, soluble factors such as growth factors and inflammatory mediators and distinct physicochemical conditions. Variation in TIME composition has been associated with clinical outcomes and therapeutic response. CIBERSORT-estimated relative immune-cell fractions and correlations among these fractions were visualized in multiple formats (Figure S6A,B). Statistically significant differences between normal and tumor tissues were observed in the CIBERSORT-estimated relative fractions of several immune-cell populations, including naive B cells, activated dendritic cells, M0 macrophages, M1 macrophages, M2 macrophages, resting mast cells, monocytes, activated NK cells, resting NK cells, activated CD4 memory T cells, resting CD4 memory T cells, follicular helper T cells, and regulatory T cells (Tregs) (Figure 3C). Spearman correlation analysis showed significant associations between candidate-gene expression and specific CIBERSORT-estimated immune-cell fractions. DNAJA4 expression was positively correlated with the estimated fractions of resting CD4 memory T cells, regulatory T cells (Tregs), and resting dendritic cells, but negatively correlated with those of activated CD4 memory T cells, follicular helper T cells, M1 macrophages, activated dendritic cells, and resting mast cells. HSPA6 expression was positively correlated with the estimated fraction of activated mast cells. LMNA expression was positively correlated with the estimated fractions of memory B cells, CD8 T cells, activated CD4 memory T cells, resting NK cells, resting mast cells, and activated mast cells while being negatively correlated with those of resting CD4 memory T cells, regulatory T cells (Tregs), and resting dendritic cells (Figure 3D).

### 3.12. Nomogram Model Prediction

Risk scores were subsequently integrated into a nomogram to visualize the multivariable regression results. The nomogram showed the contributions of the clinical variables and gene-expression signatures to the predicted 1-, 3-, and 5-year overall survival probabilities (Figure 3E). Calibration plots were used to assess agreement between the predicted and observed survival probabilities (Figure 3F). Time-dependent receiver operating characteristic curves were generated to evaluate model discrimination (Figure 3G), and decision curve analysis was performed to assess potential clinical utility (Figure 3H).

### 3.13. Predicted Drug-Sensitivity Associations and Spatial Expression Profiles of Candidate Genes

**Figure 4:**
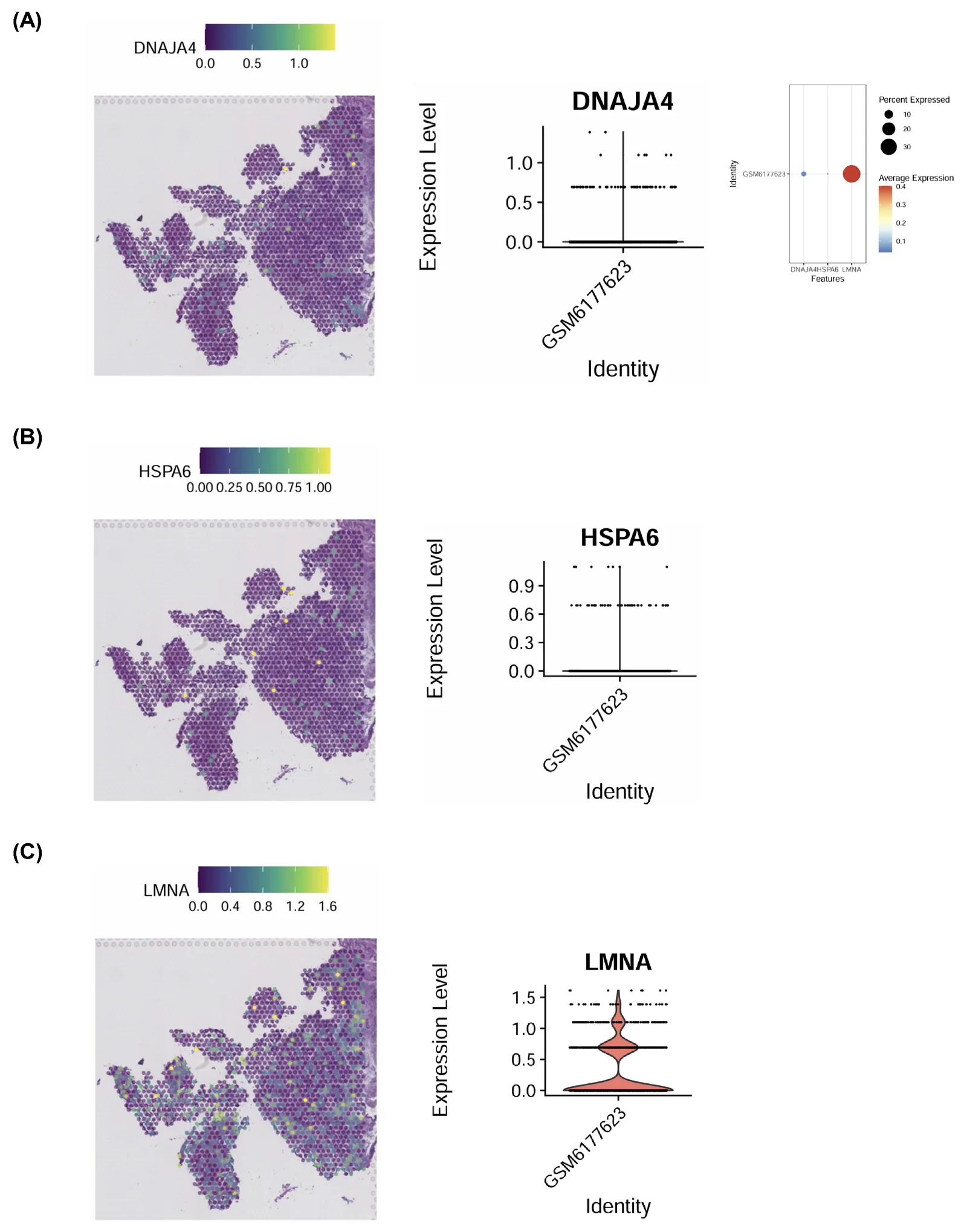
Spatial expression patterns of DNAJA4, HSPA6, and LMNA in EC. (**A**–**C**) Spatial transcriptomic maps (**left**) and corresponding violin and dot plots (**right**) showing the distribution and expression levels of DNAJA4 (**A**), HSPA6 (**B**), and LMNA (**C**) within the analyzed cross-sectional EC tissue section (n = 1).

Surgery, with adjuvant chemotherapy when clinically indicated, remains a standard treatment approach for EC. In this study, GDSC reference data were used to generate exploratory computational estimates of drug sensitivity. The oncoPredict R package was used to estimate IC_50_ values from each tumor transcriptomic profile and to examine associations between candidate-gene expression and predicted drug response. DNAJA4 expression was significantly correlated with the predicted IC_50_ values for afatinib (1032), doramapimod (1042), cytarabine (1006), vinblastine (1004), Wee1 inhibitor (1046), and gefitinib (1010) (Figure S6C). HSPA6 expression was significantly correlated with the predicted IC_50_ values for AZD7762 (1022), doramapimod (1042), cisplatin (1005), docetaxel (1007), gefitinib (1010), and PLX-4720 (1036) (Figure S6D). LMNA expression was significantly correlated with the predicted IC_50_ values for navitoclax (1011), Wee1 inhibitor (1046), nilotinib (1013), cytarabine (1006), doramapimod (1042), and vorinostat (1012) (Figure S6E). Finally, spatial mapping showed the distribution and expression levels of DNAJA4, HSPA6, and LMNA in the analyzed spatial transcriptomic dataset (Figure 4A–C).

### 3.14. Expression and Functional Assessment of Candidate Genes in EC Cells

**Figure 5:**
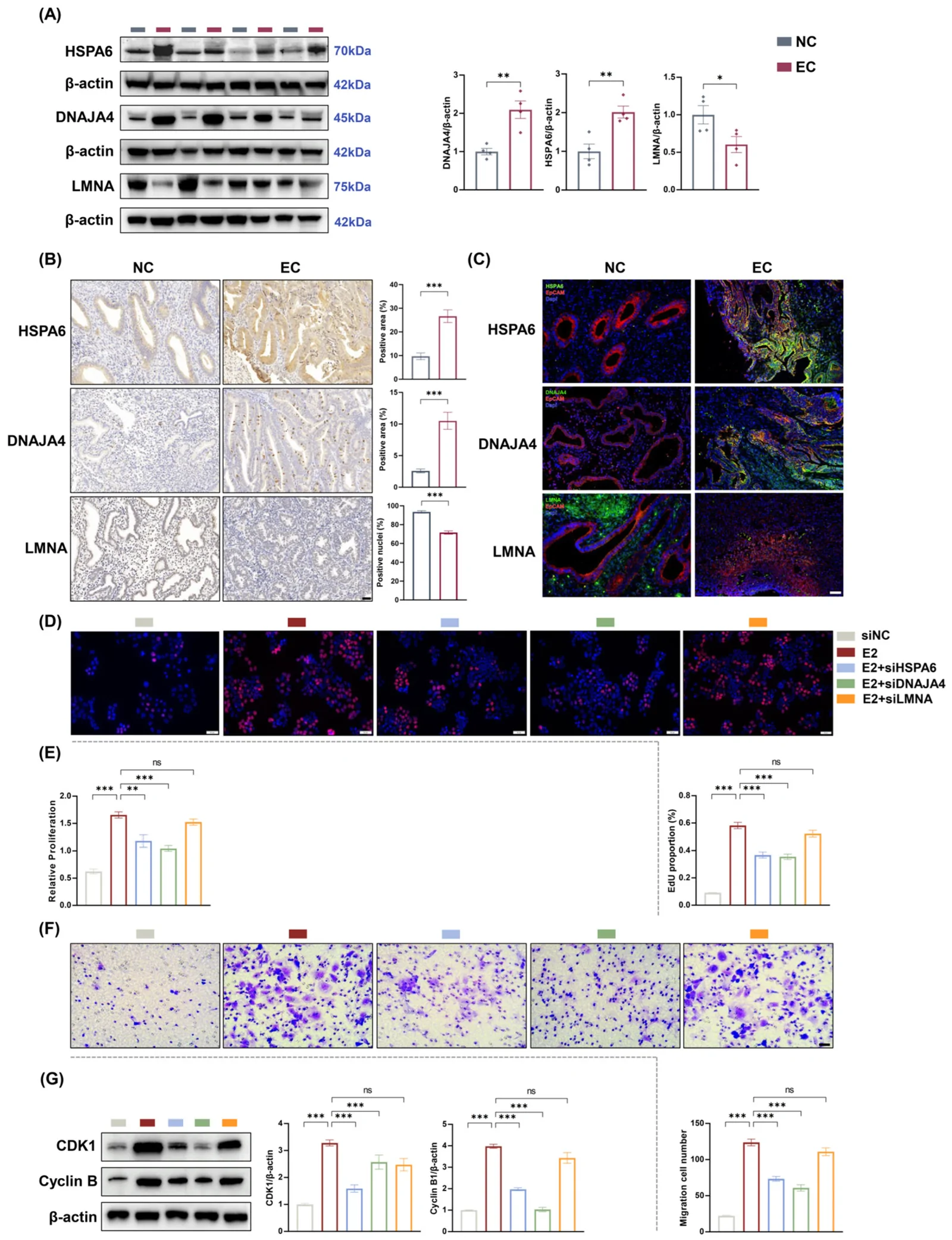
Expression and functional assessment of DNAJA4, HSPA6, and LMNA in EC. (**A**) Western blot analysis of DNAJA4, HSPA6, and LMNA expression in NC and EC tissues. The NC and EC tissues were obtained from different individuals and analyzed as independent, unpaired samples (n = 4 per group). (**B**) Representative immunohistochemical (IHC) images and quantitative analysis of protein expression in NC and EC tissues (n = 4 per group; scale bar, 100 μm). Six microscopic fields were randomly selected from each sample for quantification. (**C**) IF images showing the localization of DNAJA4, HSPA6, and LMNA in tumor epithelial cells (n = 4; scale bar, 50 μm). (**D**–**F**) Functional assays in Ishikawa cells (n = 3): Ishikawa cells were transfected with 20 nM siRNA using a commercial transfection reagent. After 1 h, the cells were treated with 10 nM 17β-estradiol (E2) for 48 h. Vehicle-treated and negative-control siRNA-transfected cells were included as controls. (**D**) EdU incorporation assay (red) showing the effects of siRNA-mediated gene knockdown on proliferation (scale bar, 50 μm). For quantification, six randomly selected microscopic fields were analyzed per sample, and the mean value was used for statistical analysis. (**E**) CCK-8 assay of cell growth and (**F**) Transwell assay of migration following gene knockdown. (**G**) Western blot analysis and quantification of the cell-cycle proteins CDK1 and Cyclin B following knockdown. Data were obtained from three independent biological experiments (n = 3). Data are presented as the mean ± SEM. * *p* < 0.05, ** *p* < 0.01, and *** *p* < 0.001; ns, not significant.

Initially, the protein expression of the candidate genes was examined in endometrial cancer tissues and independent normal control tissues. Western blotting and immunohistochemical analyses showed higher DNAJA4 and HSPA6 protein levels in tumor tissues than in normal control tissues (Figure 5A,B). IF further showed elevated expression of both proteins in tumor epithelial cells (Figure 5C). To assess their functional relevance under estrogen stimulation, each candidate gene was knocked down with siRNA in E2-treated Ishikawa cells (Figure S6F). EdU incorporation and CCK-8 assays showed reduced proliferation, whereas Transwell assays showed reduced migration following DNAJA4 or HSPA6 knockdown (Figure 5D–F). To examine associated cell-cycle changes, the levels of CDK1 and Cyclin B were measured. Both proteins were reduced following DNAJA4 or HSPA6 knockdown, consistent with decreased cell-cycle activity (Figure 5G). LMNA protein levels were significantly lower in human EC tissues than in normal control tissues (Figure 5A,B). IF analysis showed weak LMNA staining in tumor epithelial cells (Figure 5C). Following LMNA knockdown (Figure S6F), proliferation and cell-cycle protein levels showed small, nonsignificant decreases (Figure 5E–G).

## 4. Discussion

EC is characterized by marked molecular, cellular, and inflammatory microenvironmental heterogeneity, which complicates biological interpretation and therapeutic translation [6,7,18]. In this study, we combined high-resolution spatial and single-cell transcriptomic analyses with MR and functional experiments. Using this framework, we prioritized DNAJA4, HSPA6, and LMNA as candidate molecular regulators associated with MM0, a putative stemness-associated malignant epithelial transcriptional subpopulation. These candidates may have context-dependent associations with tumor-related phenotypes. Together, these analyses examined associations among malignant epithelial transcriptional states, their spatial microenvironment, and tumor-related phenotypes.

The progression of EC is shaped by cellular microenvironments that support malignant cell expansion [8,18,19]. In our cross-sectional analyses, MM0 was characterized as a putative stemness-associated transcriptional subpopulation with enhanced inferred hypoxia- and JAK–STAT-related pathway activity and appeared at an early position along the inferred cell-state differentiation trajectory. This pattern is consistent with roles reported in other tumor types for hypoxic and inflammatory signaling in stemness-associated programs [20,21,22], but it does not demonstrate functional self-renewal. Antigen-presentation genes, including HLA-A, HLA-B, and HLA-C, showed higher expression toward the opposite end of the inferred ordering than in MM0. Although HLA downregulation has been described as an immune-evasion mechanism selected during tumor progression [23,24], our cross-sectional data cannot determine when this feature arises. The observed pattern therefore suggests only an association between the MM0 stemness-related transcriptional state and lower MHC-I expression.

One question raised by our analysis concerns the context-dependent associations of the molecular chaperones DNAJA4 and HSPA6 with EC susceptibility and established-tumor phenotypes. HSPA6 is a human stress-inducible member of the HSP70 family that redistributes from the cytoplasm to nuclear structures following thermal stress [25]. In lung cancer cell models, HSPA6 deletion reduced cellular survival and proliferation and increased sensitivity to Actinidia chinensis root extract (acRoots) [26]. In addition, interaction with HSPB6 has been reported to downregulate HSPA6, thereby suppressing the JNK-JUND signaling axis and reducing colorectal cancer cell proliferation [27]. In contrast, the relationship between DNAJA4 and cancer remains poorly characterized. As a member of the human DnaJ/Hsp40 co-chaperone family, DNAJA4 may participate in HSP70-dependent proteostasis [28]. Mechanistic studies show that DnaJ interacts with the ATPase and substrate-binding regions of DnaK (Hsp70) and modulates its ATPase activity and substrate handling [29], although the specific substrates and functions of DNAJA4 in EC remain unknown. DNAJA4 hypermethylation has been reported in pediatric rhabdomyosarcoma [30].

The apparently divergent DNAJA4 findings should be interpreted across distinct evidentiary and disease-stage contexts. MR uses genetic variants as instrumental variables to estimate the relationship between a genetically proxied exposure and disease susceptibility under specific assumptions; it does not directly measure gene function after a tumor has formed [31]. In our analysis, DNAJA4 expression genetically predicted from whole-blood eQTL data was inversely associated with EC risk. In contrast, DNAJA4 protein abundance was higher in established EC tissues, and siRNA-mediated DNAJA4 knockdown in E2-stimulated Ishikawa cells reduced proliferation and migration as well as CDK1 and Cyclin B expression. These observations address different estimands: the MR result pertains to population-level disease susceptibility, whereas tissue expression and knockdown assays characterize an established tumor state and a specific cell model. Accordingly, elevated DNAJA4 protein abundance in EC tissue does not establish that DNAJA4 contributes to disease initiation and may instead represent a secondary adaptive response to the proteostatic demands of rapidly proliferating tumor cells. Likewise, the knockdown phenotype supports a functional contribution to proliferation and migration in this specific model but does not negate the inverse genetic association or establish that this effect generalizes across EC contexts.

One possible explanation is that DNAJA4 exerts stage- and context-dependent effects. Under normal endometrial conditions or before tumor establishment, HSP70-associated co-chaperone activity may help maintain proteostasis, limit the accumulation of misfolded proteins, and support cellular stress responses and genome maintenance [32,33]. After malignant transformation, however, EC cells exposed to estrogen-driven proliferation and increased proteotoxic stress may exploit chaperone networks to sustain stress tolerance and survival; such adaptive use of normally cytoprotective chaperone functions has been described more broadly in cancer [34,35,36,37]. DNAJA4 could therefore contribute to homeostatic protection in a nonmalignant context while becoming part of an adaptive chaperone dependency in established tumors, with this balance further shaped by the oncogenic state and tumor microenvironment [38,39]. This model is biologically plausible but remains hypothetical: the current data do not demonstrate a stage-specific functional switch, identify DNAJA4 clients, or establish direct stabilization of cell-cycle proteins. Longitudinal models and rescue experiments across additional EC cell lines and relevant in vivo settings will be required to test this interpretation.

Another mechanistic question of interest concerns LMNA, which encodes a key structural component of the nuclear lamina. Although MR analysis suggested a potential causal association between genetically predicted LMNA expression and increased EC risk, our tissue and cellular assays showed that LMNA was significantly downregulated in human EC tissues, and that targeted LMNA knockdown did not significantly inhibit cell proliferation in a two-dimensional in vitro epithelial cell model. The absence of a significant proliferative effect indicates that an MR association does not establish a direct proliferation-promoting role in this cell model. However, available evidence suggests that LMNA may have context-dependent roles in tumor initiation and progression [40]. LMNA expression has been reported to be upregulated in high-grade prostate cancer [41]. In contrast, reduced lamin A/C expression has been associated with more invasive phenotypes in prostate cancer cell models [42], while clinical data have linked reduced lamin A and lamin C to lymph-node positivity and poorer disease-specific survival, respectively [43]. Furthermore, experiments have shown that LMNA is downregulated in ovarian cancer. Moderate knockdown of LMNA increases the nuclear plasticity of tumor cells, thereby promoting their migratory potential. However, more extensive LMNA knockdown induces cellular damage in ovarian cancer cells and limits their migratory capacity [44]. The resulting mechanical plasticity may facilitate invasion in three-dimensional environments without necessarily increasing proliferation in two-dimensional culture. Such context dependence may partly account for the modest LMNA-knockdown effects observed here, although this explanation remains speculative. Furthermore, distinct LMNA isoforms and post-translational modifications influence disease progression. For instance, DNA damage-induced ATR activation can promote nuclear-envelope rupture through lamin A/C phosphorylation, creating a potentially targetable vulnerability in homologous-recombination-deficient cells [45]. Taken together, LMNA expression may more closely reflect tumor-associated structural stability, alterations in nuclear architecture and the nucleoskeleton, or broader changes in cellular state. Whether LMNA directly contributes to EC progression remains uncertain and requires further mechanistic and functional validation.

The translational implications of these analyses remain exploratory. Cancer immunotherapy, including immune-checkpoint blockade, adoptive cellular therapy, and cancer vaccines, has broadened the therapeutic landscape; however, its efficacy can be constrained by tumor-microenvironment heterogeneity, immune-evasion mechanisms, and treatment resistance [46]. In this context, the observed associations between candidate-gene expression and computationally estimated immune-cell fractions may generate hypotheses for future immunotherapy-oriented studies. The prognostic nomogram incorporating candidate-gene expression signatures and clinical variables showed preliminary predictive performance for overall survival (OS) within the development cohort. Separately, an exploratory oncoPredict/GDSC analysis generated hypotheses regarding potential drug–response associations. Although chemotherapy remains part of the management of advanced EC and treatment responses vary [47], molecular profiling has been proposed as a means of identifying actionable pathway alterations and refining treatment selection in poorly differentiated EC [48]. In our exploratory analysis, candidate-gene expression profiles were associated with predicted responses to agents such as Wee1 inhibitors and afatinib. These exploratory findings may inform future studies of drug response and repurposing that integrate candidate-gene expression with pharmacogenomic data.

Several limitations warrant consideration. The limited sample size, absence of multi-patient spatial replication, and cross-sectional design restrict the generalizability and temporal interpretation of the findings. MR supports genetic prioritization but does not establish tumor-intrinsic causality. Moreover, blood-derived eQTLs cannot be assumed to directly reflect tissue-specific gene regulation in endometrial or tumor tissue, which further limits the interpretation of the MR findings. Functional validation was confined to E2-stimulated Ishikawa cells in two-dimensional culture, and immune-cell associations were based on computational deconvolution without perturbation-based validation. The modest LMNA-knockdown phenotype should therefore be interpreted within the context of this restricted model. Future studies using larger independent cohorts and complementary approaches—including additional EC cell lines, rescue or overexpression experiments, three-dimensional and in vivo models, and immune-cell co-cultures—are needed to validate these findings and clarify the context-dependent role of LMNA.

## 5. Conclusions

In conclusion, this integrated multi-omics analysis prioritized DNAJA4, HSPA6, and LMNA as candidate genes showing potential causal associations with EC risk. Functional assays in the E2-stimulated Ishikawa model further supported DNAJA4 and HSPA6 as candidate functional genes, whereas the functional role of LMNA remains to be clarified. MM0 was characterized as a putative stemness-associated transcriptional subpopulation, and the analyses delineated an inferred cell-state differentiation trajectory and cross-sectional spatial associations. These bioinformatic findings do not establish true temporal or clonal evolution in vivo but provide hypotheses for future longitudinal, lineage-resolved, mechanistic, and translational studies in EC.

## Data Availability

The original contributions presented in this study are included in the article and Supplementary Materials. Further inquiries can be directed to the corresponding authors.

## Supplementary Materials

The following supporting information can be downloaded at: https://www.mdpi.com/article/10.3390/cells15171559/s1, Figure S1: Quality control and preprocessing of scRNA-Seq data; Figure S2: Single-cell transcriptomic annotation and characterization of malignant epithelial subpopulations in EC; Figure S3: Single-cell and spatial assessment of MM0-associated proliferation-related transcriptional activity; Figure S4: Pseudotemporal dynamics, spatial organization, and intercellular associations within the EC microenvironment; Figure S5: Leave-one-out sensitivity analyses of the Mendelian randomization estimates; Figure S6: Immune infiltration, predicted drug sensitivity, and validation of siRNA-mediated gene knockdown in endometrial cancer; Table S1: Clinical characteristics of the single-cell and spatial transcriptomics samples; Table S2: Heterogeneity analysis of Mendelian randomization estimates for candidate genes associated with EC; Table S3: MR instrument strength and sensitivity tests for DNAJA4, HSPA6, and LMNA.

## Abbreviations

The following abbreviations are used in this manuscript:

AUCell	AUC-based gene-set enrichment scoring algorithm
CCK-8	Cell Counting Kit-8
CIBERSORT	Cell-type Identification by Estimating Relative Subsets of RNA Transcripts
Coloc	Colocalization analysis (R package)
CopyKAT	Copy number Karyotyping of Tumors
CSC	Cancer stem cell
CytoTRACE	Cellular Trajectory Reconstruction Analysis using gene Counts and Expression
DCA	Decision curve analysis
DoubletFinder	Doublet detection algorithm
E2	17β-Estradiol
EC	Endometrial cancer
EdU	5-Ethynyl-2′-deoxyuridine
eQTL	Expression quantitative trait locus
ER	Endoplasmic reticulum
GDSC	Genomics of Drug Sensitivity in Cancer
GWAS	Genome-wide association study
HLA	Human leukocyte antigen
HSP70	Heat shock protein 70
irGSEA	Individualized Gene Set Enrichment Analysis
IVW	Inverse-variance weighted
MHC	Major histocompatibility complex
MISTy	Multiview Intercellular SpaTial modeling
MR	Mendelian randomization
OS	Overall survival
PCA	Principal component analysis
PDOs	Patient-derived organoids
PROGENy	Pathway RespOnse GENerator
RCTD	Robust Cell-Type Decomposition
ROC	Receiver operating characteristic
RRA	Robust Rank Aggregation
scRNA-Seq	Single-cell RNA sequencing
ssGSEA	Single-sample Gene Set Enrichment Analysis
stLearn	Spatial transcriptomics analysis Python package
TIME	Tumor immune microenvironment
UCell	UCell enrichment scoring algorithm
UMAP	Uniform Manifold Approximation and Projection
