# Supplementary material for: Integration of Spatial Transcriptomics and Mendelian Randomization Identifies Candidate Molecular Regulators of Endometrial Cancer Progression
Source: Cells. 2026 Aug 28;15(17):1559. doi: 10.3390/cells15171559 (PMC13564818; doi:10.3390/cells15171559)
Supplement: Supplementary file 1 [file cells-15-01559-s001.zip › cells-4463944-supplementary.pdf]

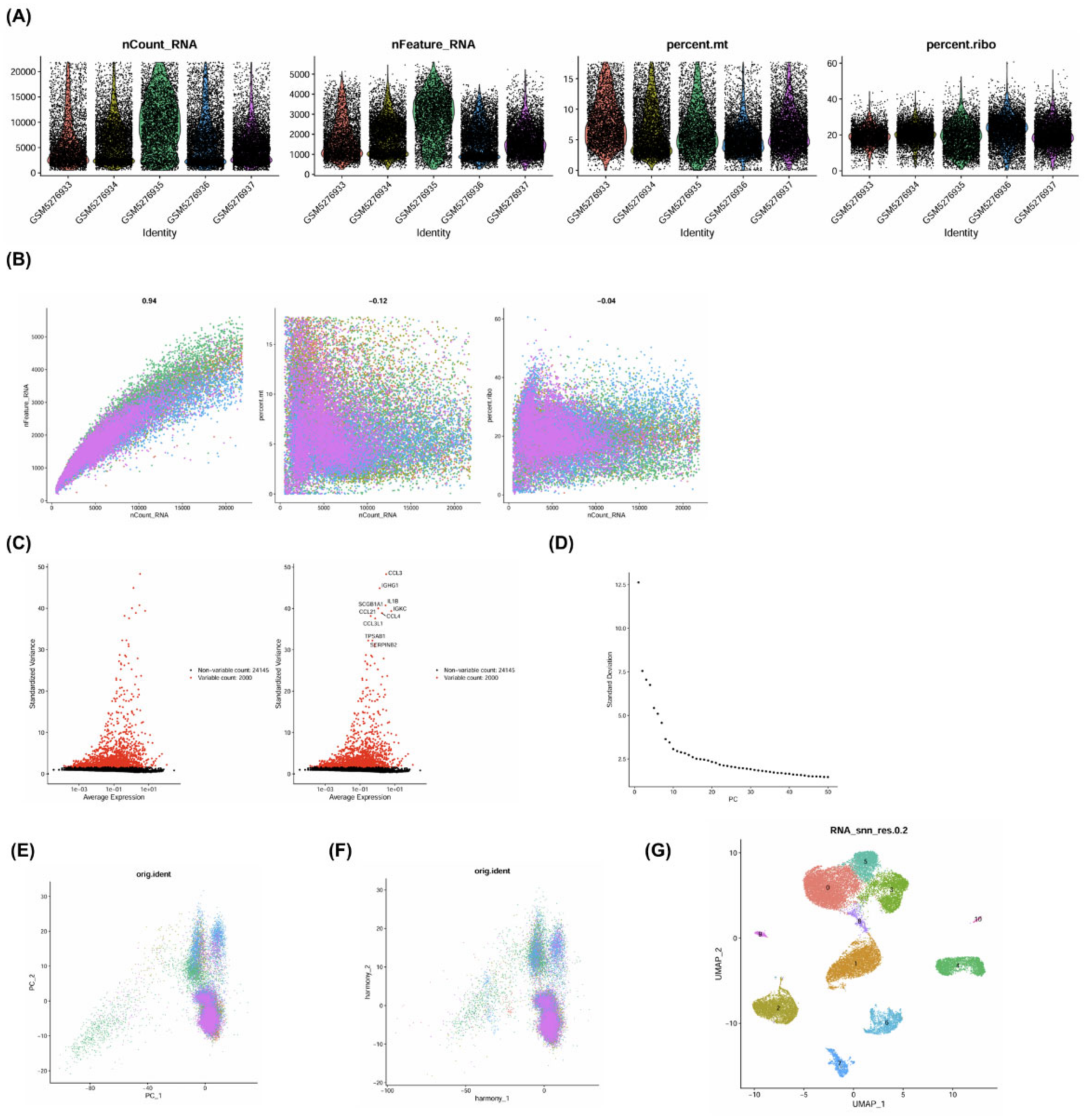

Figure S1. Quality control and preprocessing of scRNA-seq data.

(A) Violin plots displaying the quality control (QC) metrics for each sample, including the number of detected genes (nFeature_RNA), total UMI counts (nCount_RNA), and the percentage of mitochondrial (percent.mt) and ribosomal (percent.ribo) transcripts. (B) Scatter plots showing the correlation between UMI counts and detected genes/mitochondrial content, used to identify and remove low-quality cells and doublets. (C) Variance plots identifying the top 2,000 highly variable genes (red dots) used for downstream dimensionality reduction. (D) Elbow plot visualizing the standard deviation of each principal component (PC); the first 30 PCs were selected for further analysis. (E-F) PCA plots before (E) and after (F) batch effect correction using the Harmony algorithm, showing improved integration across samples. (G) UMAP visualization of the initial 11 cell clusters following unsupervised clustering (resolution = 0.2).

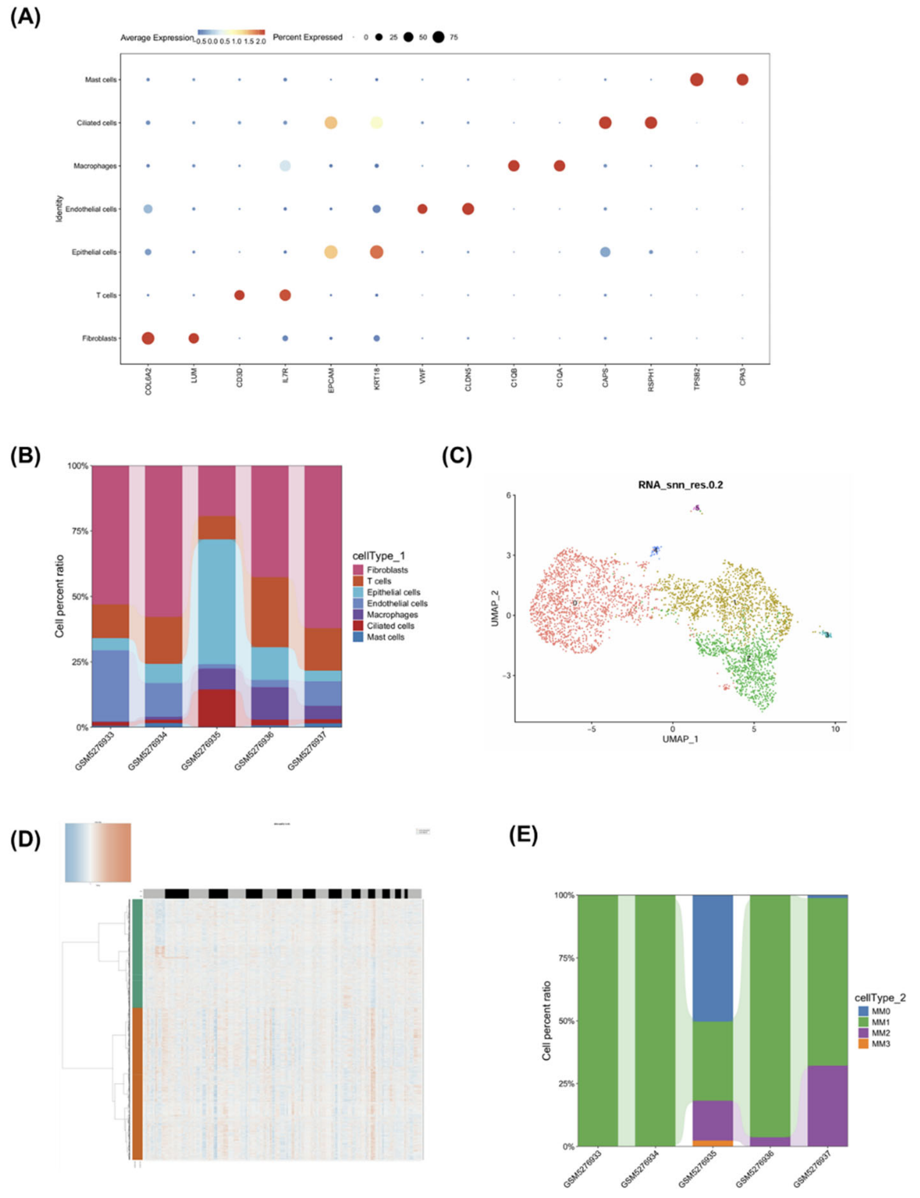

Figure S2. Single-cell transcriptomic annotation and characterization of malignant epithelial subpopulations in EC.

(A) Dot plot showing the expression of canonical marker genes across identified cell lineages. The dot size represents the percentage of cells expressing the gene, and the color intensity indicates the average expression level. (B) Bar plot illustrating the relative proportion of cell types across different patients. (C) Sub-clustering of the epithelial cell population. (D) Heatmap of large-scale chromosomal copy number variations (CNVs) inferred by CopyKAT to distinguish malignant (aneuploid) from non-malignant (diploid) epithelial cells. (E) Compositional analysis of MM subpopulations across samples.

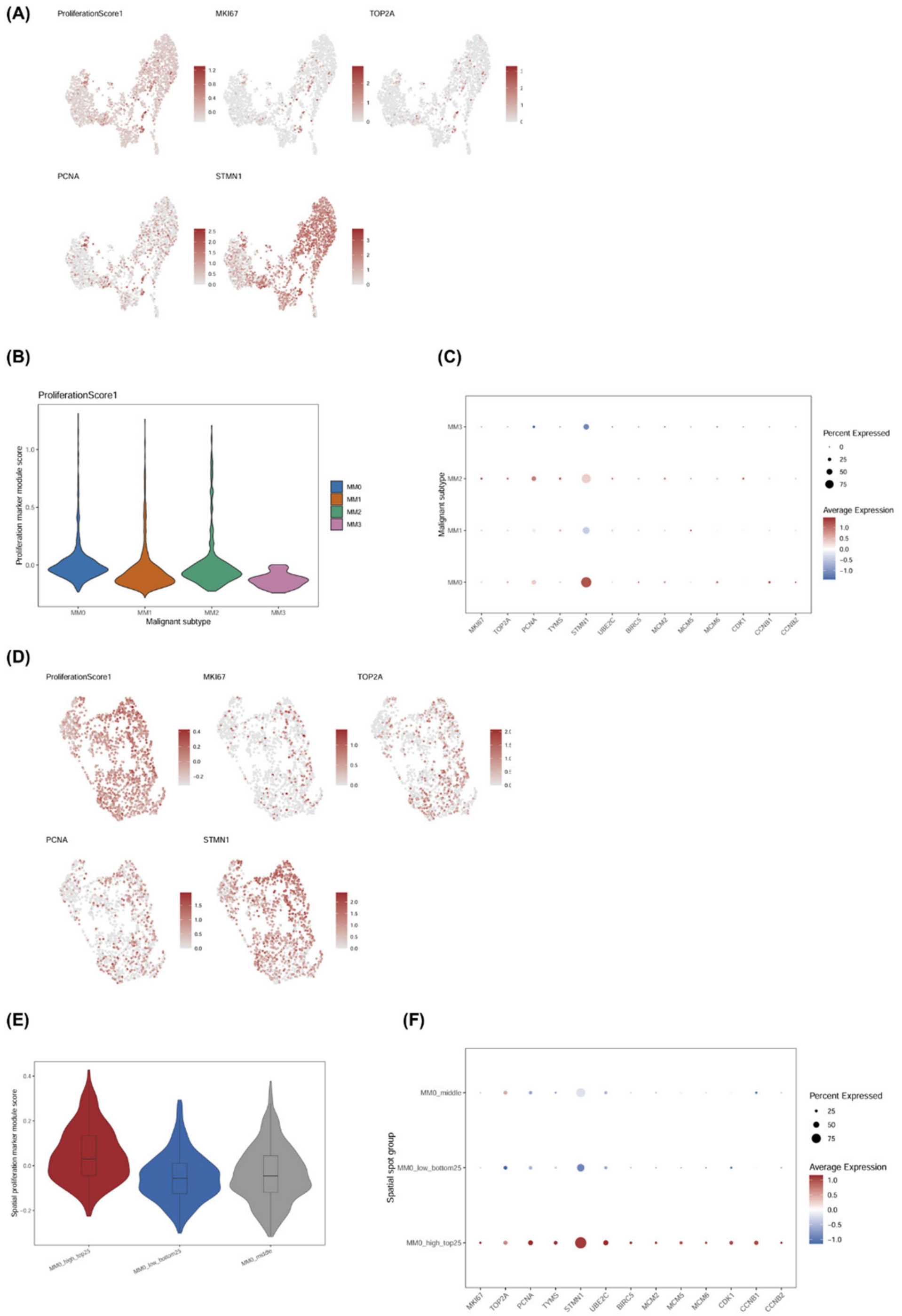

Figure S3. Single-cell and spatial assessment of MM0-associated proliferation-related transcriptional activity.

(A) UMAP feature plot showing the distribution of proliferation-related module scores across malignant epithelial cells. (B) Violin plots showing the distribution of proliferation-related module scores across the MM0–MM3 malignant epithelial subpopulations. (C) Dot plot

showing the expression of 13 proliferation-related marker genes across MM0–MM3. Dot size represents the percentage of cells expressing each gene, and color intensity indicates the average expression level. (D) Feature plot showing the distribution of proliferation-related module scores across spatial transcriptomic spots. (E) Violin plots comparing proliferation-related module scores among spatial spots stratified according to RCTD-derived MM0 weights into the top quartile (MM0_high_top25), middle 50% (MM0_middle), and bottom quartile (MM0_low_bottom25). (F) Dot plot showing the expression of the 13 proliferation-related marker genes across the corresponding MM0-weight strata. Dot size represents the percentage of spots expressing each gene, and color intensity indicates the average expression level.

Module scores were calculated using the non-regressed log-normalized expression data. These analyses reflect proliferation-related transcriptional activity rather than direct measurements of cell proliferation or cell-cycle progression. The single-cell panels summarize 2,266 malignant epithelial cells pooled across five tumors and do not provide patient-level inference. The spatial analyses were based on one cross-sectional EC tissue section (biological n = 1); individual spots represent observational units rather than independent biological replicates, and the spatial findings should therefore be considered exploratory.

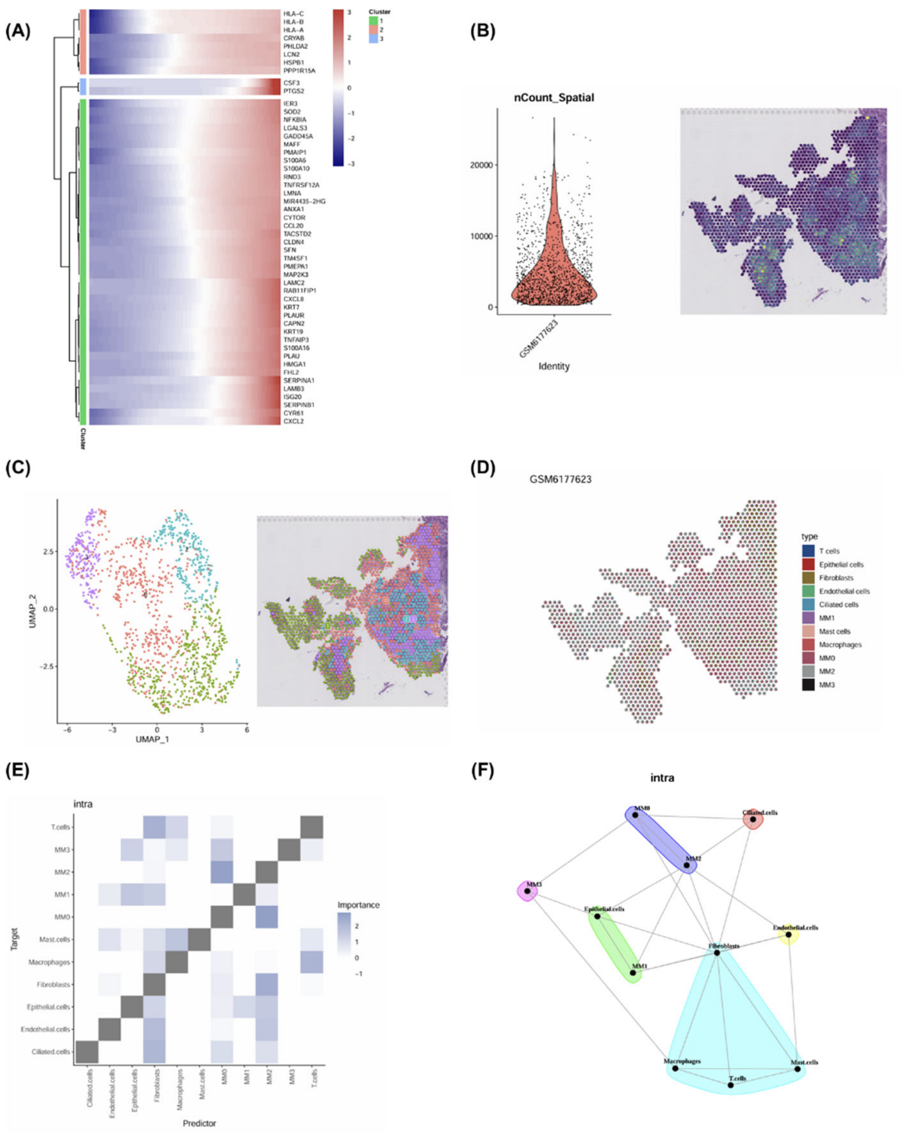

Figure S4. Pseudotemporal dynamics, spatial organization, and intercellular interactions within the EC microenvironment.

(A) Heatmap showing dynamic expression patterns of key genes along the pseudotime continuum. (B) Quality control of spatial transcriptomics data (GSM6177623), showing the distribution of UMI counts (nCount_Spatial) per spot and its corresponding spatial heatmap. (C) UMAP and spatial mapping of Bayesian-clustered spots within the tissue architecture. (D) Spatial distribution of deconvolved cell types (RCTD) across the tissue section, illustrating the topographic organization of epithelial, stromal, and immune populations. (E-F) MISTy intercellular interaction heatmap and network diagram showing the spatial crosstalk between MM subpopulations and other stromal/immune components.

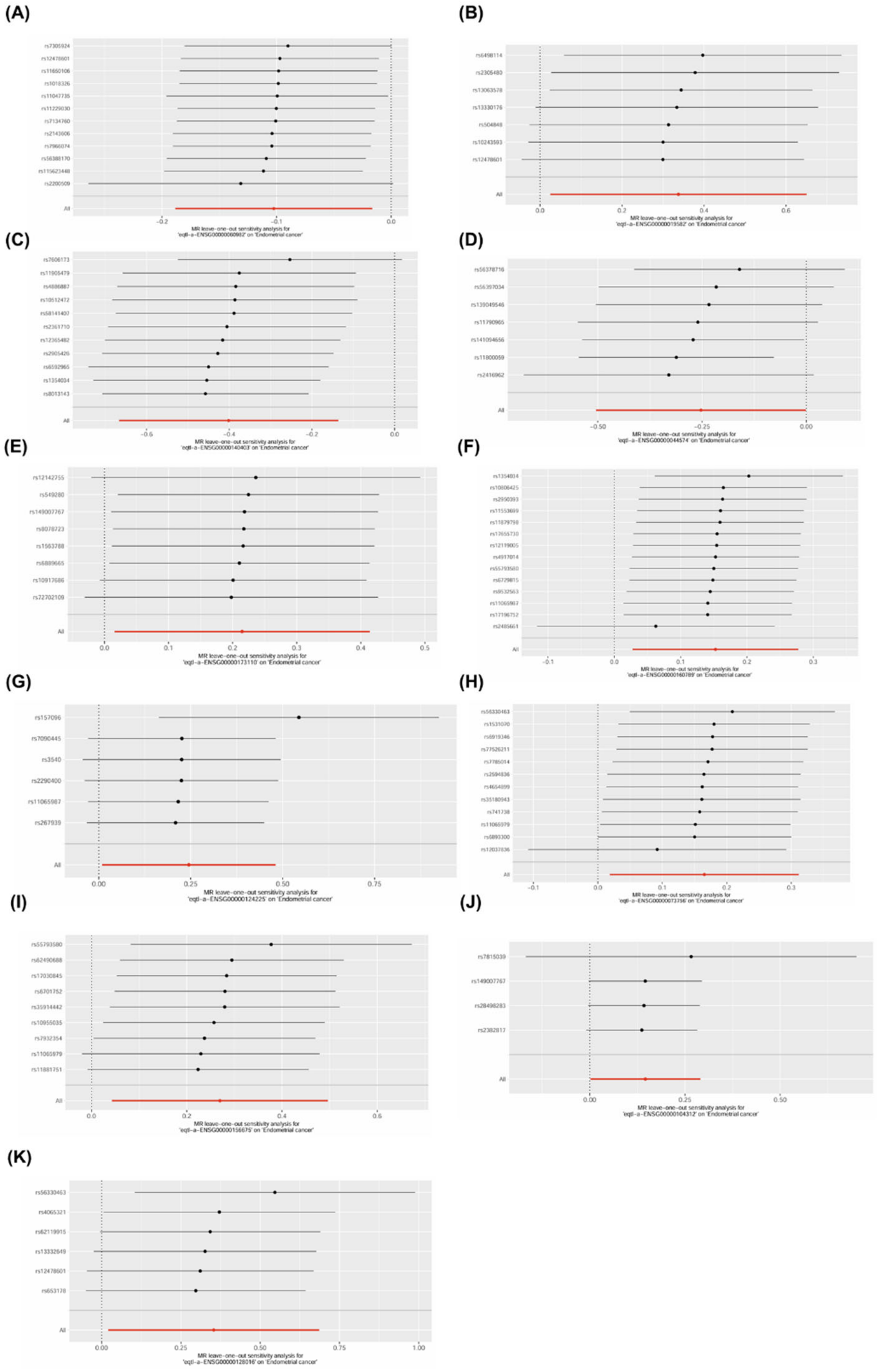

Figure S5. Leave-one-out sensitivity analyses of the Mendelian randomization estimates. (A–K) Leave-one-out analyses performed for each investigated gene to assess the robustness of the corresponding MR estimates.

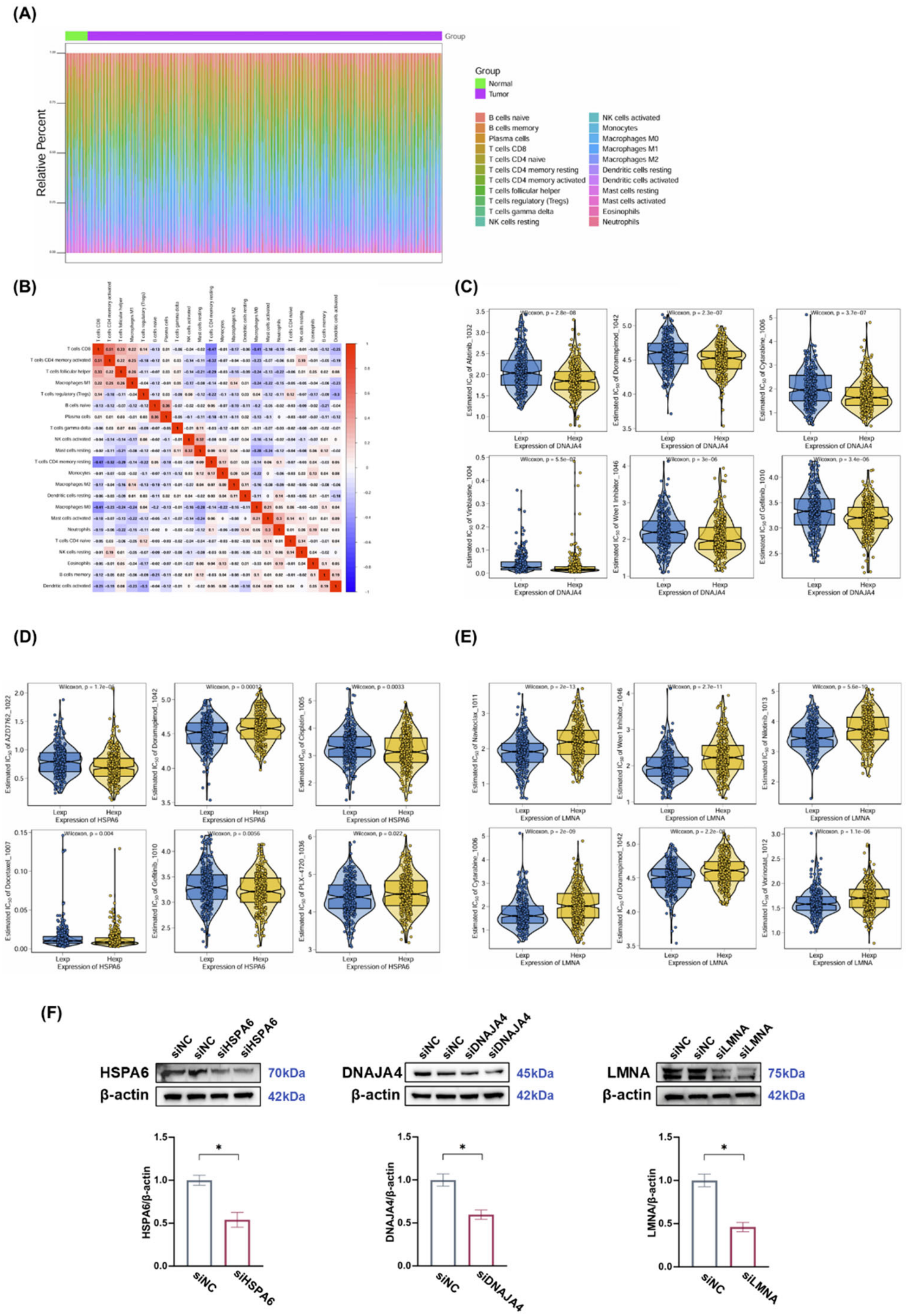

Figure S6. Immune infiltration, predicted drug sensitivity, and validation of siRNA-mediated gene knockdown in endometrial cancer.

(A–B) CIBERSORT deconvolution showing the relative proportions of 22 immune-infiltrating cell types and their correlation matrix in EC. (C–E) Boxplots showing the predicted sensitivity (IC50) to various chemotherapeutic and targeted agents according to the expression levels of the three key genes. (F) Western blot analysis of HSPA6, DNAJA4, and LMNA protein levels in Ishikawa cells 48 h after transfection with the corresponding siRNAs. The results showed significantly reduced

expression of the target proteins, confirming efficient knockdown for subsequent functional assays. Western blot quantification data are presented as the mean ± SEM from two independent biological experiments (n = 2); $P < 0.05$.

Table S1

!Sample_geo_accession	!Sample_source_name_ch1	!Sample_characteristics_ch1	!Sample_characteristics_ch1	!Sample_characteristics_ch1	!Sample_characteristics_ch1	!Sample_characteristics_ch1	!Sample_characteristics_ch1
GSM5276933	Tumor of the endometrium	race_ethnicity: African American	tissue: Tumor	tumor_site: Endometrium	histology: Endometrioid	metastatic status: Primary/Non-metastatic	patient: 1
GSM5276934	Tumor of the endometrium	race_ethnicity: Caucasian	tissue: Tumor	tumor_site: Endometrium	histology: Endometrioid	metastatic status: Primary/Non-metastatic	patient: 2
GSM5276935	Tumor of the endometrium	race_ethnicity: Caucasian	tissue: Tumor	tumor_site: Endometrium	histology: Endometrioid	metastatic status: Primary/Non-metastatic	patient: 3
GSM5276936	Tumor of the endometrium	race_ethnicity: Caucasian	tissue: Tumor	tumor_site: Endometrium	histology: Endometrioid	metastatic status: Primary/Non-metastatic	patient: 4
GSM5276937	Tumor of the endometrium	race_ethnicity: Caucasian	tissue: Tumor	tumor_site: Endometrium	histology: Endometrioid	metastatic status: Primary/Non-metastatic	patient: 5
Title	Source name	Organism	Characteristics				
NYU_UCEC3_Vis 10x Genomics Visium	Spatial transcriptomics of patient primary endometrial carcinoma	Homo sapiens	cancer type: Endometrial carcinoma	organ: Endometrium	method: 10x Genomics Visium		

Table S1: single cell and Spatial transcriptomics clinical

Table S2

	id.exposure	id.outcome	outcome	exposure	method	Q	Q_df	Q_pval
BCAT1	ENSG00000060982	GCST90018838	Endometrial cancer	eqtl-a-ENSG00000060982	MR Egger	6.2945914	10	0.78994
	ENSG00000060982	GCST90018838	Endometrial cancer	eqtl-a-ENSG00000060982	Inverse variance weighted	7.905304	11	0.72176
CD74	ENSG00000019582	GCST90018838	Endometrial cancer	eqtl-a-ENSG00000019582	MR Egger	1.4082847	5	0.9234
	ENSG00000019582	GCST90018838	Endometrial cancer	eqtl-a-ENSG00000019582	Inverse variance weighted	1.7296769	6	0.94281
DNAJA4	ENSG00000140403	GCST90018838	Endometrial cancer	eqtl-a-ENSG00000140403	MR Egger	6.7003702	9	0.66828
	ENSG00000140403	GCST90018838	Endometrial cancer	eqtl-a-ENSG00000140403	Inverse variance weighted	11.970008	10	0.28707
HSPA5	ENSG00000044574	GCST90018838	Endometrial cancer	eqtl-a-ENSG00000044574	MR Egger	6.4247884	5	0.26705
	ENSG00000044574	GCST90018838	Endometrial cancer	eqtl-a-ENSG00000044574	Inverse variance weighted	7.4534658	6	0.28094
HSPA6	ENSG00000173110	GCST90018838	Endometrial cancer	eqtl-a-ENSG00000173110	MR Egger	0.4816402	6	0.99805
	ENSG00000173110	GCST90018838	Endometrial cancer	eqtl-a-ENSG00000173110	Inverse variance weighted	0.5546768	7	0.99922
LMNA	ENSG00000160789	GCST90018838	Endometrial cancer	eqtl-a-ENSG00000160789	MR Egger	9.6566626	12	0.64605
	ENSG00000160789	GCST90018838	Endometrial cancer	eqtl-a-ENSG00000160789	Inverse variance weighted	10.401886	13	0.66079
PMEPA1	ENSG00000124225	GCST90018838	Endometrial cancer	eqtl-a-ENSG00000124225	MR Egger	0.4765696	4	0.97574
	ENSG00000124225	GCST90018838	Endometrial cancer	eqtl-a-ENSG00000124225	Inverse variance weighted	4.801784	5	0.44055
PTGS2	ENSG00000073756	GCST90018838	Endometrial cancer	eqtl-a-ENSG00000073756	MR Egger	9.568191	10	0.47916
	ENSG00000073756	GCST90018838	Endometrial cancer	eqtl-a-ENSG00000073756	Inverse variance weighted	9.6998151	11	0.55757
RAB11FIP1	ENSG00000156675	GCST90018838	Endometrial cancer	eqtl-a-ENSG00000156675	MR Egger	4.8570281	7	0.67741
	ENSG00000156675	GCST90018838	Endometrial cancer	eqtl-a-ENSG00000156675	Inverse variance weighted	7.0421418	8	0.53209
RIPK2	ENSG00000104312	GCST90018838	Endometrial cancer	eqtl-a-ENSG00000104312	MR Egger	0.1879639	2	0.9103
	ENSG00000104312	GCST90018838	Endometrial cancer	eqtl-a-ENSG00000104312	Inverse variance weighted	0.8569111	3	0.83581
ZFP36	ENSG00000128016	GCST90018838	Endometrial cancer	eqtl-a-ENSG00000128016	MR Egger	0.8820399	4	0.92712
	ENSG00000128016	GCST90018838	Endometrial cancer	eqtl-a-ENSG00000128016	Inverse variance weighted	2.8061203	5	0.72985

Table S2. Heterogeneity analysis of Mendelian randomization estimates for candidate genes associated with EC.

Cochran's Q test was used to evaluate heterogeneity across SNP- specific causal estimates for the 11 candidate genes in the MR analyses Heterogeneity was assessed using both MR-Egger and inverse-variance weighted methods. Q, Q_df, and Q_pval denote Cochran's Q statistic, the corresponcing degrees of freedom, and the heterogeneity P value, respectively. A Q_pval > 0.05 indicates no statistically significant evidence of heterogeneity.

Table S3

Supplementary Table S3. MR instrument strength and sensitivity tests for DNAJA4

Summary of instrument strength, pleiotropy and heterogeneity

**Tool SNP count**	11
**Mean F statistic**	30.6359336946645
**MR-Egger intercept**	0.0854492538357203
**MR-Egger intercept SE**	0.0372235578560486
**MR-Egger intercept P**	0.0473413535471891
**Cochran Q - MR Egger**	6.70037022551431
**Cochran Q df - MR Egger**	9
**Cochran Q P - MR Egger**	0.668282749645191
**Cochran Q - IVW**	11.9700082893666
**Cochran Q df - IVW**	10
**Cochran Q P - IVW**	0.287068751471261

MR-Egger pleiotropy full results

Gene	id.exposure	id.outcome	outcome	exposure	egger_intercept	se	pval
DNAJA4	ENSG00000140403	GCST90018838	Endometrial cancer	eqtl-a-ENSG00000140403	0.0854492538357203	0.03722355785 60486	0.047341353547 1891

Cochran heterogeneity full results

Gene	id.exposure	id.outcome	outcome	exposure	method	Q	Q_df	Q_pval
DNAJA4	ENSG00000140403	GCST90018838	Endometrial cancer	eqtl-a-ENSG00000140403	MR Egger	6.7003702255 1431	9	0.66828274964 5191
DNAJA4	ENSG00000140403	GCST90018838	Endometrial cancer	eqtl-a-ENSG00000140403	Inverse variance weighted	11.970008289 3666	10	0.28706875147 1261

Instrument SNP-level F statistics

Gene	SNP	Chr	Position	Effect_allele	Other_allele	Beta_exposure	SE_exposure	P_exposure	N_exposure	Beta_outcome	SE_outcome	P_outcome	N_outcome	F_statistic
DNAJA4	rs10512472	17	33884804	C	T	-0.0830729	0.0155085	8.47090868220201e-08	26609	0.0425	0.0295	0.1492	301841	28.6932111558208
DNAJA4	rs11905479	20	55977759	A	G	-0.0790435	0.0148427	1.00730270453096e-07	26493	0.0595	0.0374	0.1116	301841	28.3600182135315
DNAJA4	rs12365482	11	16612936	T	A	-0.0591779	0.0132737	8.25733682001747e-06	26494	0.0094	0.0308	0.7601	301841	19.8762798805177
DNAJA4	rs1354034	3	56849749	C	T	-0.0653887	0.012238	9.14218488704808e-08	26609	-0.0041	0.0253	0.8697	301841	28.5485798194848
DNAJA4	rs2361710	17	78111877	C	T	0.050121	0.0118962	2.51779287373585e-05	26225	-0.0169	0.0254	0.5057	301841	17.7510047704748
DNAJA4	rs2905426	19	19478022	T	G	0.0523191	0.0123941	2.42789555497189e-05	22897	0.0003	0.0263	0.9895	301841	17.819295810896
DNAJA4	rs4886887	15	77898203	T	C	-0.0613781	0.0137786	8.40504416852668e-06	26492	0.0386	0.0286	0.1765	301841	19.843427821478
DNAJA4	rs58141407	8	21791772	T	C	0.083382	0.0169731	8.99684006121017e-07	26494	-0.0506	0.0422	0.2308	301841	24.1336092029622
DNAJA4	rs6592965	7	50427982	A	G	0.0794403	0.0119459	2.93494522226979e-11	26609	-0.0091	0.0258	0.723	301841	44.222572367955
DNAJA4	rs7606173	2	60725451	C	G	-0.112089	0.0121487	2.79898131963436e-20	26494	0.111	0.0309	0.0003203	301841	85.126812871712
DNAJA4	rs8013143	14	23494277	G	A	0.0626592	0.0131745	1.97469487940933e-06	26271	0.0336	0.0326	0.3018	301841	22.6204587264771

Supplementary Table S3. MR instrument strength and sensitivity tests for HSPA6

Summary of instrument strength, pleiotropy and heterogeneity

Tool SNP count	8
Mean F statistic	67.7572345510488
MR-Egger intercept	-0.00646193330690508
MR-Egger intercept SE	0.0239106918136382
MR-Egger intercept P	0.796021079160776
Cochran Q - MR Egger	0.481640224904233
Cochran Q df - MR Egger	6
Cochran Q P - MR Egger	0.998054794660255
Cochran Q - IVW	0.554676843633809
Cochran Q df - IVW	7
Cochran Q P - IVW	0.999220630423267

MR-Egger pleiotropy full results

Gene	id.exposure	id.outcome	outcome	exposure	egger_intercept	se	pval
HSPA6	ENSG00000173110	GCST90018838	Endometrial cancer	eqtl-a-ENSG00000173110	-0.00646193330690508	0.0239106918136382	0.796021079160776

Cochran heterogeneity full results

Gene	id.exposure	id.outcome	outcome	exposure	method	Q	Q_df	Q_pval
HSPA6	ENSG00000173110	GCST90018838	Endometrial cancer	eqtl-a-ENSG00000173110	MR Egger	0.481640224904233	6	0.998054794660255
HSPA6	ENSG00000173110	GCST90018838	Endometrial cancer	eqtl-a-ENSG00000173110	Inverse variance weighted	0.554676843633809	7	0.999220630423267

Instrument SNP-level F statistics

Gene	SNP	Chr	Position	Effect_allele	Other_allele	Beta_exposure	SE_exposure	P_exposure	N_exposure	Beta_outcome	SE_outcome	P_outcome	N_outcome	F_statistic
HSPA6	rs10917686	1	161658490	T	C	-0.105627	0.0145825	4.36918063385288e-13	14262	-0.0389	0.037	0.2924	301841	52.4669622758292
HSPA6	rs12142755	1	161481743	G	A	-0.188906	0.0131482	8.26418441715798e-47	14259	-0.0345	0.0304	0.2565	301841	206.423387646833
HSPA6	rs149007767	7	50370254	T	C	0.102508	0.0166593	7.60308769982229e-10	13100	0.0179	0.0357	0.6161	301841	37.8618667237381
HSPA6	rs1563788	6	43308363	T	C	0.0620861	0.0129295	1.57249763156399e-06	14263	0.0116	0.0269	0.6666	301841	23.0581945492743
HSPA6	rs549280	4	74971196	A	G	-0.0541088	0.0123626	1.20539670507711e-05	14263	-0.0007	0.0254	0.9784	301841	19.1565002550093
HSPA6	rs6889665	5	23532643	C	T	-0.147446	0.0346826	2.12441813451933e-05	14263	-0.0478	0.0774	0.5365	301841	18.0735180106725
HSPA6	rs72702109	1	161575093	G	T	0.160132	0.0124755	1.03609600704048e-37	11158	0.0428	0.033	0.1948	301841	164.755656762779
HSPA6	rs8078723	17	38166879	C	T	0.0551568	0.0122535	6.75709461347849e-06	14263	0.0091	0.025	0.716	301841	20.261790184255

Supplementary Table S3. MR instrument strength and sensitivity tests for LMNA

Summary of instrument strength, pleiotropy and heterogeneity

**Tool SNP count**	14
**Mean F statistic**	131.495101364361
**MR-Egger intercept**	-0.0102445702879036
**MR-Egger intercept SE**	0.0118672635223705
**MR-Egger intercept P**	0.404916626023008
**Cochran Q - MR Egger**	9.65666262446516
**Cochran Q df - MR Egger**	12
**Cochran Q P - MR Egger**	0.646052826205523
**Cochran Q - IVW**	10.4018857447923
**Cochran Q df - IVW**	13
**Cochran Q P - IVW**	0.660786673401603

MR-Egger pleiotropy full results

Gene	id.exposure	id.outcome	outcome	exposure	egger_intercept	se	pval
LMNA	ENSG00000160789	GCST90018838	Endometrial cancer	eqtl-a-ENSG00000160789	-0.0102445702879036	0.0118672635223705	0.404916626023008

Cochran heterogeneity full results

Gene	id.exposure	id.outcome	outcome	exposure	method	Q	Q_df	Q_pval
LMNA	ENSG00000160789	GCST90018838	Endometrial cancer	eqtl-a-ENSG00000160789	MR Egger	9.65666262446516	12	0.646052826205523
LMNA	ENSG00000160789	GCST90018838	Endometrial cancer	eqtl-a-ENSG00000160789	Inverse variance weighted	10.4018857447923	13	0.660786673401603

Instrument SNP-level F statistics

Gene	SNP	Chr	Position	Effect_allele	Other_allele	Beta_exposure	SE_exposure	P_exposure	N_exposure	Beta_outcome	SE_outcome	P_outcome	N_outcome	F_statistic
LMNA	rs10806425	6	90926612	A	C	0.06371	0.0122661	2.0582114979253e-07	31300	-0.03	0.0296	0.3108	301841	26.9775314942518
LMNA	rs11065987	12	112072424	G	A	-0.0910625	0.0122337	9.78362954784597e-14	31185	-0.0431	0.0312	0.1678	301841	55.4068532247462
LMNA	rs11553699	12	122216910	G	A	0.0797146	0.0190382	2.82670182957468e-05	30031	-0.0403	0.0474	0.3955	301841	17.5316976672347
LMNA	rs11879798	19	44281898	A	G	0.0647117	0.0123021	1.43899736935702e-07	31355	-0.0113	0.0286	0.6932	301841	27.669869584263
LMNA	rs12119005	1	156241354	A	G	-0.0813472	0.013125	5.71847201363453e-10	27811	-0.0037	0.0391	0.9249	301841	38.4137401053751
LMNA	rs1354034	3	56849749	C	T	0.187931	0.0121478	5.49667424889203e-54	31684	-0.0041	0.0253	0.8697	301841	239.332448070618
LMNA	rs17196752	20	48887268	T	C	0.0865998	0.014832	5.26404993062149e-09	31684	0.0482	0.0329	0.1435	301841	34.0905764121265
LMNA	rs17655730	11	270715	C	T	0.0842259	0.0140201	1.88521115125378e-09	31569	0.0043	0.0348	0.9023	301841	36.0901841549607
LMNA	rs2485661	1	156083496	C	T	-0.899708	0.0255052	1e-200	28469	-0.2138	0.08	0.00749	301841	1244.35901208232
LMNA	rs2950393	12	57036023	A	G	0.0663014	0.0127734	2.09840832229887e-07	31569	-0.0171	0.0264	0.5165	301841	26.9421576565229
LMNA	rs4917014	7	50305863	G	T	0.0549703	0.0127886	1.72040224260263e-05	31300	0.0079	0.0259	0.7617	301841	18.4760958541911
LMNA	rs55793580	9	127009395	T	C	-0.088695	0.015833	2.11889775094869e-08	31569	-0.0203	0.032	0.5256	301841	31.3813666142701

Gene	SNP	Chr	Position	Effect_allele	Other_allele	Beta_exposure	SE_exposure	P_exposure	N_exposure	Beta_outcome	SE_outcome	P_outcome	N_outcome	F_statistic
LMNA	rs6729815	2	60723672	C	T	0.0487233	0.0119543	4.58564045367465e-05	31569	0.0214	0.0271	0.4296	301841	16.6121211326348
LMNA	rs9532563	13	41160270	C	T	-0.0744081	0.0141511	1.45468848142459e-07	31569	-0.0406	0.0339	0.231	301841	27.647765047536

Table S3. MR instrument strength and sensitivity tests for DNAJA4, HSPA6, and LMNA.

The table summarizes instrument strength, MR-Egger pleiotropy tests, Cochran's Q heterogeneity tests, SNP-level instrument characteristics, and full sensitivity-analysis results. Abbreviations: MR, Mendelian randomization; SNP, single-nucleotide polymorphism; Chr, chromosome; SE, standard error; IVW, inverse-variance weighted; df, degrees of freedom; eQTL, expression quantitative trait locus; Q, Cochran's Q statistic. Beta_exposure and Beta_outcome denote the effect estimates for the exposure and outcome, respectively; N_exposure and N_outcome denote the corresponding sample sizes.
